# Fumagillin Attenuates Spinal Angiogenesis, Neuroinflammation, and Pain in Neuropathic Rats after Chronic Constriction Injury

**DOI:** 10.3390/biomedicines9091187

**Published:** 2021-09-10

**Authors:** Zhi-Hong Wen, Shi-Ying Huang, Hsiao-Mei Kuo, Chao-Ting Chen, Nan-Fu Chen, Wu-Fu Chen, Kuan-Hao Tsui, Hsin-Tzu Liu, Chun-Sung Sung

**Affiliations:** 1 Department of Marine Biotechnology and Resources, National Sun Yat-sen University, Kaohsiung 804201, Taiwan; wzh@mail.nsysu.edu.tw (Z.-H.W.); hsiaomeikuo@gmail.com (H.-M.K.); cct337@gmail.com (C.-T.C.); ma4949@adm.cgmh.org.tw (W.-F.C.); 2 Institute of BioPharmaceutical Sciences, National Sun Yat-sen University, Kaohsiung 804201, Taiwan; 3 College of Ocean Food and Biological Engineering, Jimei University, Xiamen 361021, China; johnhuang@jmu.edu.cn; 4 Center for Neuroscience, National Sun Yat-sen University, Kaohsiung 804201, Taiwan; 5 Department of Surgery, Division of Neurosurgery, Kaohsiung Armed Forces General Hospital, Kaohsiung 802301, Taiwan; chen06688@gmail.com; 6 Institute of Medical Science and Technology, National Sun Yat-sen University, Kaohsiung 804201, Taiwan; 7 Department of Neurosurgery, Kaohsiung Chang Gung Memorial Hospital and Chang Gung University College of Medicine, Kaohsiung 833401, Taiwan; 8 Department of Obstetrics and Gynecology, Kaohsiung Veterans General Hospital, Kaohsiung 813414, Taiwan; khtsui60@gmail.com; 9 Institute of Clinical Medicine, School of Medicine, National Yang Ming Chiao Tung University, Taipei 112304, Taiwan; 10 Department of Medical Research, Hualien Tzu Chi Hospital, Buddhist Tzu Chi Medical Foundation, Hualien 970473, Taiwan; hsintzuliu@tzuchi.com.tw; 11 Department of Anesthesiology, Division of Pain Management, Taipei Veterans General Hospital, Taipei 112201, Taiwan; 12 School of Medicine, National Yang Ming Chiao Tung University, Taipei 112304, Taiwan

**Keywords:** angiogenesis, astrocytes, fumagillin, neuroinflammation, neuropathic pain

## Abstract

Introduction: Angiogenesis in the central nervous system is visible in animal models of neuroinflammation and bone cancer pain. However, whether spinal angiogenesis exists and contributes to central sensitization in neuropathic pain remains unclear. This study analyzes the impact of angiogenesis on spinal neuroinflammation in neuropathic pain. Methods: Rats with chronic constriction injury (CCI) to the sciatic nerve underwent the implantation of an intrathecal catheter. Fumagillin or vascular endothelial growth factor-A antibody (anti-VEGF-A) was administered intrathecally. Nociceptive behaviors, cytokine immunoassay, Western blot, and immunohistochemical analysis assessed the effect of angiogenesis inhibition on CCI-induced neuropathic pain. Results: VEGF, cluster of differentiation 31 (CD31), and von Willebrand factor (vWF) expressions increased after CCI in the ipsilateral lumbar spinal cord compared to that in the contralateral side of CCI and control rats from post-operative day (POD) 7 to 28, with a peak at POD 14. Tumor necrosis factor-α (TNF-α), interleukin-1β (IL-1β), and IL-6 concentrations, but not IL-10 levels, also increased in the ipsilateral spinal cord after CCI. Fumagillin and anti-VEGF-A reduced CCI-induced thermal hyperalgesia from POD 5 to 14 and mechanical allodynia from POD 3 to 14. Fumagillin reduced CCI-upregulated expressions of angiogenic factors and astrocytes. Furthermore, fumagillin decreased TNF-α and IL-6 amounts and increased IL-10 levels at POD 7 and 14, but not IL-1β concentrations. Conclusions: Fumagillin significantly ameliorates CCI-induced nociceptive sensitization, spinal angiogenesis, and astrocyte activation. Our results suggest that angiogenesis inhibitor treatment suppresses peripheral neuropathy-induced central angiogenesis, neuroinflammation, astrocyte activation, and neuropathic pain.

## 1. Introduction

The central nervous system (CNS) is composed of various cells, including neurons and glial cells. It consumes a large amount of oxygen and energy under normal (20% of the total body oxygen demand at rest) and pathological conditions. The CNS is highly vascularized, as evidenced by the high blood flow in the normal human adult brain (50 mL/min/100 g) or in the monkey spinal cord (10–20 mL/min/100 g) at rest [1]. Vascular cells, in close contact with neurons and glial cells, form the neurovascular unit [2]. Additionally, higher blood flow and metabolic rate in gray matter, compared to those in white matter, implies that neurons and glia cells require profuse blood flow to support their normal functions. Autoregulation, oxygen, carbon dioxide, neurovascular coupling, neuron-astrocyte regulation, metabolic by-products, neurotransmitters, and ion channels control cerebral blood flow [3,4,5,6]. Astrocytes, the most abundant glial cells in the CNS, extend processes in contact with blood vessels and synapses and play supportive roles in regulating blood flow and synaptic transmission, maintaining synaptic homeostasis in physiological conditions, and distributing metabolism substrates in pathological situations, including stroke, seizure, neuroinflammation, and central sensitization in chronic pain [7,8,9,10,11,12].

Lesions or dysfunctions of the nervous system result in nociceptive hypersensitization, neuronal/synaptic plasticity, and neuroinflammation. They upregulate inflammatory mediator production, immune cell infiltration, and neuronal–glial activation in the CNS, contributing to central sensitization and the development of neuropathic pain [13]. The elevated cellular/synaptic activities and increased nociceptive signal transduction observed during central sensitization require an adequate blood flow to the CNS to supply the necessary oxygen and nutrients. Transcranial doppler sonography reveals alterations in cerebral blood flow in acute pain processing in patients with fibromyalgia. Additionally, magnetic resonance spectroscopy studies revealed diminished brain energy reserve in patients with migraine attacks [14,15]. Angiogenesis, a process promoting the formation of new blood vessels from preexisting vessels, occurs in physiological (including reproduction, tissue repair, and wound healing) and pathological (including arthritis, diabetic retinopathy, and cancer) conditions. It is characterized by an elevated metabolic demand [16,17,18]. Central neuroinflammation in conjunction with angiogenesis, i.e., increased blood vessel densities and vascular endothelial growth factor (VEGF) expression in the spinal cord, has been shown in human multiple sclerosis and rodent models of experimental autoimmune encephalomyelitis (EAE) and seizure [19,20,21,22]. Chronic constriction injury (CCI) of the sciatic nerve generates long-term neuropathic pain.

Moreover, it induces a regional increase of blood flow in multiple brain structures that parallel the nociceptive behavioral changes in rats, as evidenced using technetium-99m [99mTc] neuroimaging examination [23,24]. A correlation between these phenomena suggests that angiogenesis in the CNS is critical for developing central sensitization to pain. Intrathecal administration of VEGF-A antibodies or VEGF receptor inhibitors attenuates the pain in rat models of bone metastasis from breast cancer and CCI [25,26]. However, the relationship between increased blood flow in the CNS and central nociceptive sensitization in neuropathic pain remains unclear.

Fumagillin, a mycotoxin produced by *Aspergillus fumigatus*, has antibiotic and anti-angiogenic properties. It covalently binds and inhibits methionine aminopeptidase type 2 (MetAP2) to hinder endothelial cell proliferation, angiogenesis, and tumor-induced neovascularization [27] and has been used in conjunction with anti-tumor therapy such as 5-fluorouracil and the dendritic cell tumor vaccine to inhibit tumor and prevent metastasis [28]. MetAP2 overexpression occurs in human neurofibromatosis (NF) 1-associated pilocytic astrocytoma tumors, mouse Nf1 optic pathway glioma tumors, and the cerebrospinal fluid of *Nf1*+/−^GFAP^ conditional knockout mice [29]. Fumagillin not only significantly reduces *Nf1*−/*−* astrocyte proliferation in vitro [29] but also suppresses its formation by reducing the branching point numbers of capillary-like structures in an in vitro model consisting of human brain microvascular endothelial cells, pericytes, and astrocytes plated on a gel matrix [30].

The following points regarding the link between angiogenesis and neuropathic pain require clarification: (1) Is there angiogenesis, and are angiogenesis factors upregulated in the spinal cord in neuropathic pain? (2) Is spinal angiogenesis involved in neuroinflammation development (e.g., regulation of pro-inflammatory/anti-inflammatory cytokine homeostasis and astrocyte activation) and pain processing in neuropathic pain? (3) Have anti-angiogenic factors any therapeutic effect on neuropathic pain? We hypothesize that spinal angiogenesis occurs during the development/maintenance of neuropathic pain and that treatment with fumagillin will suppress the peripheral neuropathy-induced central angiogenesis that leads to neuropathic pain.

## 2. Materials and Methods

### 2.1. Animal Preparation

All experiments and animal use were approved by the Institutional Animal Care and Use Committee of National Sun Yat-sen University (Approval No. IACUC-10447) on 1st February 2016; the use of animals conformed to the Guiding Principles in the Care and Use of Animals, published by the American Physiological Society. All efforts were exerted to minimize the number of animals used and their suffering. Adult male Wistar rats (250–285 g; BioLASCO Taiwan Co., Taipei, Taiwan) were used for all experiments. The rats were housed in plexiglass cages in a temperature-controlled (22 ± 1 °C) and 12 h light/dark-scheduled room, with free access to food and water. All operations and drug injections were performed under 2–3% isoflurane inhalation anesthesia and aseptic preparation. Post-operative care included the topical application of 10% povidone–iodine solution and intramuscular injection of cefazolin (170 mg/kg) to prevent infection, lidocaine infiltration to reduce pain, and individual housing. Rats with locomotor dysfunction after intrathecal (i.t.) catheterization and operation on post-operative day (POD) 3 were excluded from the study, and each rat was used for a single experiment only. After various drug treatments, rats that developed motor deficits or abnormal nociceptive behaviors (such as vocalizations and flaccidity) were also excluded from experiments. In our study, we also examined the gross appearance of the spinal cord after the removal of the spinal cord. The spinal cord specimen was discarded and excluded from the subsequent Western blot and immunohistochemical analyses if we found any petechiae, hematoma, or gross spinal cord, even without motor deficit. In addition, the rats for the nociceptive behavior study were also sacrificed after the completion of the experiment, and their spinal cords were examined. Only the rats without hematoma or injury in their spinal cord were included for the analysis of behavioral data. Therefore, we are confident that the observed biochemical and biological effects were not evoked by the deployment of the intrathecal catheter and intrathecal treatment.

Fumagillin and anti-VEGF antibody were delivered in 10 µL artificial cerebrospinal fluid (aCSF), which consisted of 122.7 mM Cl^−^, 21.0 mM HCO_3_^−^, 2.5 mM HPO_4_^2−^, 151.1 mM Na^+^, 0.9 mM Mg^2+^, 1.3 mM Ca^2+^, 3.5 mM dextrose, and 2.6 mM K^+^. The rats were randomly assigned to one of four groups: (i) the control group (sham operation)—the rats received aCSF i.t. injection; (ii) the chronic constriction injury (CCI) group—the rats received i.t. aCSF; (iii) the CCI + fumagillin group—the rats received i.t. fumagillin (0.1 µg/day); (iv) the CCI + anti-VEGF group— the rats received i.t. anti-VEGF-A monoclonal antibody (0.3 µg/day). All experimenters were blinded to the group allocation except for the principal investigator and the researcher who performed the sham operation. All i.t. catheters were flushed with 10 µL aCSF to consider the 3.5 µL dead volume of the i.t. catheter to ensure complete drug delivery.

### 2.2. Induction of Peripheral Neuropathy by CCI of the Sciatic Nerve

We performed either the CCI or sham operation to the right sciatic nerve of rats immediately after i.t. catheterization, as conducted in previous studies [7,31]. For the sham-operated rats (control group), the operation was performed to expose the right sciatic nerve without ligation.

### 2.3. Construction and Implantation of i.t. Catheter and i.t. Drug Injection

The i.t. catheter was prepared, and all rats were implanted with the i.t. catheter through the atlanto–occipital membrane, advancing caudally to the lumbar enlargement level of the spinal cord, for spinal drug administration, as previously described [31,32]. All rats were intrathecally injected with either aCSF (vehicle) or drugs immediately after the CCI or sham operation, as group allocated, once daily from POD 0–14.

### 2.4. Nociceptive Behavioral Assessment

#### 2.4.1. Plantar Test for Thermal Stimulation

Paw withdrawal latency (PWL, in seconds) to the radiant heat produced by an analgesiometer was applied to the right hindpaw (IITC Inc., Woodland Hills, CA, USA) to assess the positive sign of nociceptive behavior (licking or withdrawal) to thermal stimulation [7,33]. Rats were placed in compartmentalized clear plastic chambers onto an elevated glass platform, and we positioned a radiant heat source with a low-intensity heat to target the middle of the plantar surface with a cutoff time of 30 s to measure PWL. PWL was assessed from the average of three tests and separated by 3 min at baseline and different times after the i.t. injection by independent examiners who were unaware of the allocated groups.

#### 2.4.2. Von Frey Filament Test

Paw withdrawal threshold (PWT, in grams) in response to calibrated von Frey filaments (Stoelting, Wood Dale, IL, USA) was applied to the right hindpaw to assess mechanical hyperalgesia/allodynia as a positive sign of nociceptive behavior, as previously described [7,31]. The rats were placed in compartmentalized clear plastic chambers, which were set on an elevated metal mesh floor; we applied a series of von Frey filaments to the middle of the plantar region by Chaplan’s up–down method to determine the closest filament to the threshold of pain response (licking or withdrawal) [34]. PWT was also assessed from the average of three tests, separated by 3 min, by independent examiners.

#### 2.4.3. Hot-Water Immersion Test

Tail-flick latency to hot-water immersion was measured within the distal 2 cm of the tail immersed in 49 °C water at the time (seconds) of tail withdrawal due to heat hyperalgesia. The analgesic effect was expressed as the percent of maximal possible effect (% MPE) over time, and it was calculated for each dose and time point: % MPE = 100% × (measured latency − baseline latency)/(cutoff latency − baseline latency). The cutoff time for the hot-water immersion test was 20 s to minimize the possibility of thermal injury [35]. Tail-flick latency was assessed from the average of three tests, separated by 2 min, by independent examiners.

#### 2.4.4. Narrow-Beam Walking Test

This test was used to assess the motor coordination of rat gait to maintain balance to cross an elevated, narrow wooden beam (100 cm long, 4 cm wide, and 3 cm tall), which was suspended 1 m from the table. The time for the rat to use all four feet on the platform and traverse the whole length of the beam was measured, and the maximum time allowed to accomplish the task was within 2 min, in accordance with the study of Allbutt and Henderson [36]. Three successful trials were conducted, separated by 2 min, by independent examiners who were unaware of the allocated experimental groups.

#### 2.4.5. Weight Bearing Test

For the measurement of the hindpaw weight-bearing deficits (i.e., the change in hindpaw weight distribution), the rats were allowed to place their hindpaws onto the two force transducers of an incapacitance tester (Singa Technology Corporation, Taoyuan, Taiwan), as described in our previous study [7]. The naive rats distributed weight equally between both their hindlimbs. However, after inducing inflammation or injury to one hindlimb, the rats redistributed their weight to lower the weight bore by the affected hindlimb. Changes in the hindpaw weight distribution (g) of rats were expressed as the difference obtained by subtracting the weight distribution of the affected limb (ipsilateral side) from that of the normal limb (contralateral side). An average of three tests, with 2 min separation, was assessed by independent examiners.

### 2.5. Western Blotting

The rats were sacrificed under deep isoflurane anesthesia, and the dorsal part of the lumbar spinal cord enlargement was collected by exsanguination on POD 3, 7, 14, and 28. Western blotting analysis was performed as described previously [32,37]. The polyvinylidene difluoride membranes were incubated with either rabbit polyclonal anti-VEGF antibody (1:1000 dilution, catalog no. 07-1420; EMD Millipore, Bedford, MA, USA) or mouse monoclonal anti-β-actin antibody (1:5000 dilution; catalog no. A5441; Sigma-Aldrich, St Louis, MO, USA), re-incubated with horseradish peroxidase-conjugated secondary antibodies, and then measured by chemiluminescence. Densitometry was used to evaluate the density of bands relative to the background, and β-actin was used as the internal control for protein loading. Images were obtained using the UVP BioChemi Imaging System (UVP LCC, Upland, CA, USA), and relative densitometric quantification was performed using LabWorks 4.0 software (UVP LCC).

### 2.6. Immunohistochemical Assay

The immunohistochemistry protocol and quantification of images were performed as previously described [7,32]. Briefly, under deep isoflurane anesthesia (5%), the rats were transcardially perfused with cold phosphate-buffered saline (PBS) (pH 7.4) with heparin (0.2 U/mL), followed by ice-cold 4% paraformaldehyde in PBS on POD 7, 14, 21, and 28. Lumbar enlargement of the spinal cord was harvested, mounted on a tissue block, cut into 20 µm-thick sections, mounted serially onto microscope slides, and processed for immunofluorescence studies. These sections were incubated with a blocking buffer for 1 h and then incubated with either primary antibodies overnight at 4 °C: mouse monoclonal anti-glial fibrillary acidic protein antibody (GFAP; astrocytic marker, 1:200 dilution, catalog no. MAB3402; EMD Millipore, Temecula, CA, USA), rabbit polyclonal anti-von Willebrand factor antibody (vWF, 1:250 dilution, catalog no. GTX60934; GeneTex, Irvine, CA, USA), rabbit polyclonal anti-VEGF antibody (1:200 dilution, catalog no. 07-1420; EMD Millipore, Temecula, CA, USA), and mouse monoclonal anti-CD31 antibody (cluster of differentiation 31 or platelet endothelial cell adhesion molecule-1, 1:200 dilution, catalog no. ab64543; Abcam, Cambridge, MA, USA). After washing in PBS, the sections were incubated with either Alexa Fluor 488-labeled chicken anti-mouse IgG antibody (1:400 dilution, catalog no. A-21200; Molecular Probes, Eugene, OR, USA; green fluorescence) or DyLight 549-conjugated donkey anti-rabbit secondary antibody (1:400 dilution; catalog no. 711-506-152; Jackson ImmunoResearch Laboratories Inc., West Grove, PA, USA; red fluorescence).

The sections were examined under a Leica DM-6000B fluorescence microscope (Leica, Wetzlar, Germany) equipped with a CCD Spot Xplorer integrating camera (Diagnostic Instruments, Inc., Sterling Heights, MI, USA) and analyzed by SPOT software (Diagnostic Instruments Inc., USA). Image J software (National Institutes of Health, Bethesda, MD, USA) was used for pixel measurement and analysis. For quantification of immunofluorescence acquired from lamina I to lamina IV in the spinal cord dorsal horn (SCDH), four randomly selected sections were measured in each rat, and the means of 3 rats in each group were calculated. The sizes and conditions of the image captures and views were kept constant at each side of SCDH, and pixel measurement and analysis were performed with MetaVue Imaging software (Molecular Devices Corporation, Sunnyvale, CA, USA). The immunoreactivity level data are shown as the fold change relative to the control group, which was considered to represent a fold change of 1.

### 2.7. Measurement of Cytokine Concentrations in the Spinal Cord

The rats were sacrificed by decapitation under deep isoflurane anesthesia, and the spinal cord was collected by exsanguination. The dorsal part of the lumbar enlargement of the spinal cord was immediately dissected from the right and left sides on ice and flash frozen in 2 mL microcentrifuge tubes at −80 °C until processing. The tissue samples were homogenized by sonication in 1 mL ice-cold PBS in the presence of a protease inhibitor cocktail (1:100 dilution; P8340, Sigma-Aldrich, St. Louis, MO, USA) and centrifuged at 12,000× *g* for 15 min. The supernatants were collected and stored at −80 °C until analysis. Total protein was determined using a DC Protein Assay Reagents Package (catalog no. 5000116; Bio-Red Laboratories, Hercules, CA, USA), and 2 mg/mL total protein was used for the analysis. The concentrations of tumor necrosis factor-α (TNF-α), interleukin-1β (IL-1β), IL-6, and IL-10 were analyzed using a Bio-Plex Pro^TM^ Rat Cytokine and TGF-β 4-Plex Assay Kit (Bio-Rad Laboratories, Hercules, CA, USA). In brief, we added 50 µL beads to the well and washed them twice. Then, we added 50 µL of homogenates and incubated them with the antibody-coupled beads for 60 min at room temperature. After washing three times to remove unbound materials, the beads were incubated with 25 µL biotinylated detection antibodies for 30 min at room temperature. After washing away the unbound biotinylated antibodies thrice, the beads were incubated with 50 µL streptavidin–phycoerythrin for 10 min at room temperature. Following the removal of excess streptavidin–phycoerythrin after three washes, the beads were re-suspended in 125 µL of assay buffer. Finally, the beads were read on a Bio-Plex suspension array system, and the data were analyzed using the Bio-Plex Manager software.

### 2.8. Drug Preparation

The anti-VEGF-A monoclonal antibody (catalog no.05-443; Millipore, Bedford, MA, USA) was dissolved in physiological saline and prepared for i.t. injection at a concentration of 0.3 µg/10 µL. Fumagillin (F6771; Sigma-Aldrich, St Louis, MO, USA) was initially dissolved in dimethyl sulfoxide at a concentration of 0.5 mg/mL and stored at −20 °C in aliquots. This stock solution was then diluted in physiological saline for i.t. use at a concentration of 0.1 µg/10 µL.

### 2.9. Statistical Analysis

Data are expressed as means ± standard error of means (SEMs). Changes in the protein level and immunofluorescence reactivity were expressed relative to the values of the control group. For statistical analyses, the differences between groups were calculated using a one-way analysis of variance, followed by a post hoc Tukey test. We defined the statistical significance as *p* < 0.05. Statistical analyses were performed by using SPSS for Windows version 17.0 (SPSS Inc., Chicago, IL, USA).

## 3. Results

### 3.1. Animals

The experimental design is shown in the supplementary materials (Figure S1). In brief, 147 rats were surgically prepared and intrathecally catheterized. Two rats displayed locomotor dysfunctions and had not recovered on POD 3. Therefore, 145 rats were used in the study.

### 3.2. Time-Dependent Changes of Spinal VEGF Protein Levels in CCI Rats

**Figure 1:**
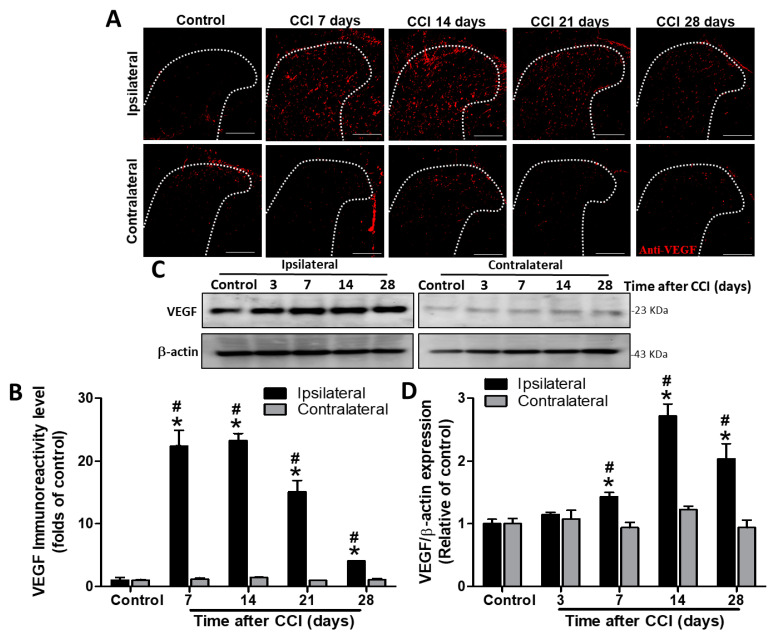
Immunohistochemistry and Western blot analyses of vascular endothelial growth factor (VEGF) expression in the rat lumbar spinal cord dorsal horn (SCDH) during the 28 days after induction of neuropathic pain by chronic constriction injury (CCI). (**A**) Representative VEGF immunofluorescence (red) in the ipsilateral SCDH of controls and CCI rats at post−operative day (POD) 7, 14, 21, and 28 (20−μm sections; magnification: 100×; scale bars: 300 μm). (**B**) Quantification of immunofluorescence reactivity in the contralateral and ipsilateral SCDHs (4 sections per rat, 3 rats per group and each time point). (**C**) Representative VEGF and β−actin Western blots in the ipsilateral spinal cord of control rats and CCI rats at POD 3, 7, 14, and 28 (*n* = 3 at each group and each time point). (**D**) Quantification of the Western blot results. The loading control was β−actin, the quantification was performed on four rats per group, and values are expressed as means ± standard error of means (SEMs). * *p* < 0.05 relative to the same side of controls; # *p* < 0.05 relative to the contralateral side of the same group at the same time point, one-way analysis of variance (ANOVA) with post hoc Tukey test.

To determine whether angiogenesis occurs in the spinal cord, we evaluated VEGF expression by Western blot and immunohistochemistry. VEGF immunoreactivity was significantly upregulated in the ipsilateral dorsal horn of the spinal cord after CCI at POD 7, 14, 21, and 28 (22.4-fold, *p* < 0.001; 23.3-fold, *p* < 0.001; 15.1-fold, *p* = 0.0017; and 4.1-fold, *p* = 0.002; respectively), but not in the contralateral side (1.2-, 1.5-, 1.0-, and 1.1-folds, respectively; all *p* > 0.05), compared to VEGF staining in controls (POD 7 after sham operation) (*n* = 3 per group; Figure 1A,B). In addition, VEGF immunoreactivity was also significantly upregulated in the ipsilateral SCDH after CCI at POD 7, 14, 21, and 28 (22.4- vs. 1.2-fold, *p* < 0.001; 23.3- vs. 1.5-fold, *p* < 0.001; 15.1- vs. 1-fold, *p* = 0.0015; and 4.1- vs. 1.1-fold, *p* < 0.001; respectively) compared to it in the contralateral side at the same time point (*n* = 3 per group; Figure 1A,B). Western blot analyses also revealed increased VEGF protein levels in the ipsilateral lumbar spinal cord dorsal part on POD 7, 14, and 28 (1.4-fold, *p* = 0.019; 2.7-fold, *p* = 0.0019; and 2.0-fold, *p* = 0.0243; respectively) but not on POD 3 (1.2-fold, *p* = 0.18) after CCI (*n* = 4 per group; Figure 1C,D) compared to that in control rats. Uncropped Western blots of VEGR and β-actin are shown in the supplementary materials (Figure S2). There was no significant difference in VEGF protein content between the contralateral spinal cord of CCI rats at POD 3, 7, 14, and 28 (1.1-, 1.0-, 1.2-, and 0.9-folds, respectively; all *p* > 0.05) and the control group (*n* = 4 per group; Figure 1C,D). Similarly, VEGF proteins were also significantly upregulated in the ipsilateral SCDH after CCI at POD 7, 14, and 28 (1.4- vs. 0.9-fold, *p* = 0.0152; 2.7- vs. 1.2-fold, *p* = 0.0029; and 2.0- vs. 0.9-fold, *p* = 0.0246; respectively), but not at POD 3 (1.2- vs. 1.1-fold, *p* = 0.684) compared to the contralateral side at the same time point (*n* = 4 per group; Figure 1C,D). These results demonstrate that CCI induces the upregulation of VEGF expression in the ipsilateral spinal cord from POD 7 to 28, with a peak at POD 14.

### 3.3. CCI Upregulates CD31 and vWF Protein Levels in the Spinal Cord in a Similar Time-Dependent Manner

**Figure 2:**
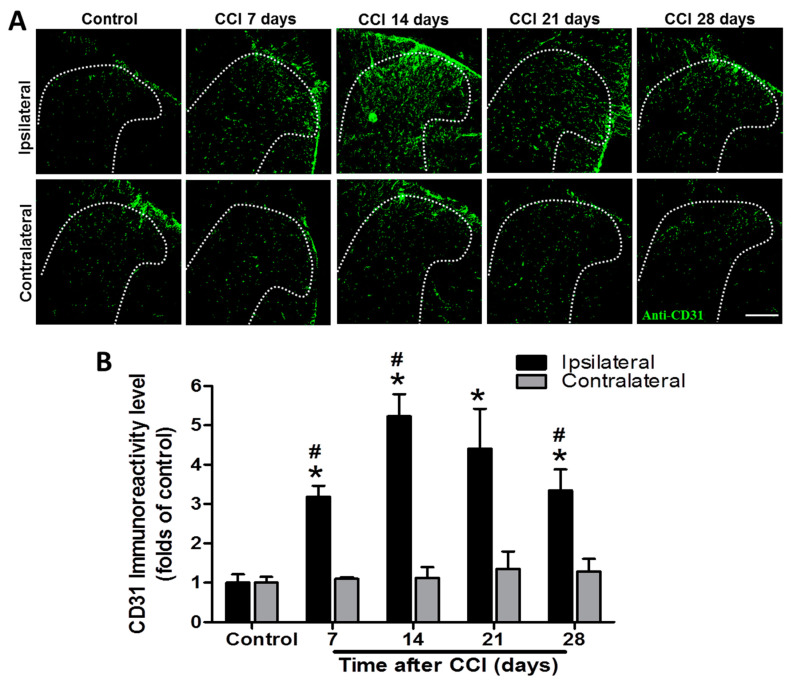
Representative cluster of differentiation 31 (CD31) immunostaining in the rat lumbar spinal cord dorsal horn (SCDH) over a 28−day period of chronic constriction injury (CCI)−induced neuropathic pain. (**A**) Representative CD31 immunofluorescence (green) in the ipsilateral SCDH of control rats and CCI rats at post−operative day (POD) 7, 14, 21, and 28 (20−μm sections; magnification: 100×; scale bars: 300 μm). (**B**) Quantification of immunofluorescence reactivity in the contralateral and ipsilateral SCDHs (4 sections per rat, 3 rats per group and each time point); values are expressed as means ± standard error of means (SEMs). * *p* < 0.05 relative to the same side of controls; # *p* < 0.05 relative to the contralateral side of the same group at the same time point, one-way analysis of variance (ANOVA) with post hoc Tukey test.

CD31 immunoreactivity was markedly increased from POD 7 to 28 after CCI, with a peak at POD 14 in the ipsilateral SCDH compared to that in the control group (3.2-fold, *p* = 0.002; 5.2-fold, *p* = 0.002; 4.4-fold, *p* = 0.031; and 3.3-fold, *p* = 0.015 at POD 7, 14, 21, and 28, respectively), while it was not affected on the contralateral side (1.1-, 1.1-, 1.3-, and 1.3-folds; all *p* > 0.05) (*n* = 3 per group; Figure 2). In addition, CD31 immunoreactivity was significantly upregulated in the ipsilateral SCDH after CCI at POD 7, 14, and 28 (3.2- vs. 1.1-fold, *p* < 0.001; 5.2- vs. 1.1-fold, *p* =0.0081; and 3.3- vs. 1.3-fold, *p* = 0.0299; respectively), but not at POD 21 (4.4- vs. 1.3-fold, *p* = 0.052) compared to the contralateral side at the same time point (*n* = 3 per group; Figure 2).

**Figure 3:**
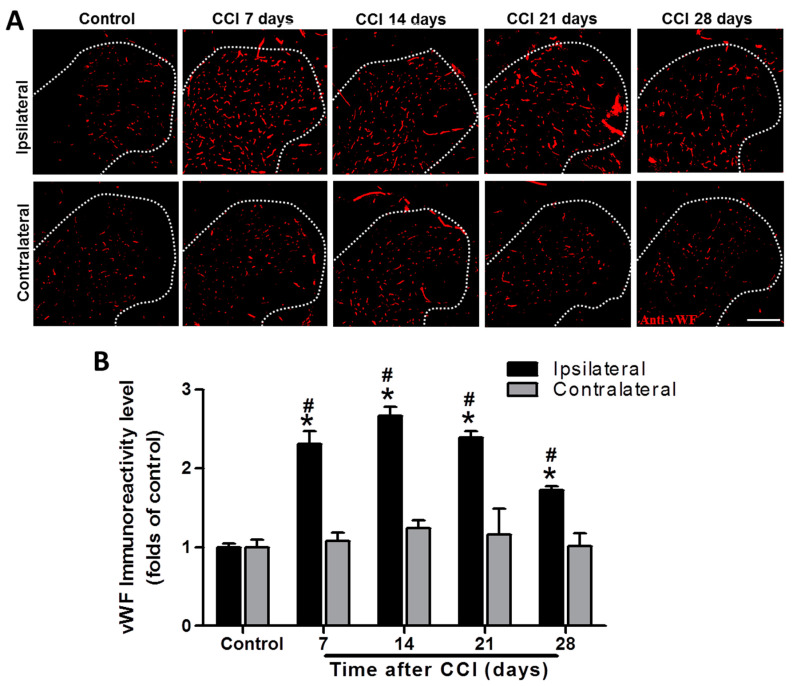
Representative von Willebrand factor (vWF) immunostaining in the rat lumbar spinal cord dorsal horn (SCDH) over a 28−day period of chronic constriction injury (CCI)−induced neuropathic pain. (**A**) Representative vWF immunofluorescence (red) in the ipsilateral SCDH of control rats and CCI rats at post−operative day (POD) 7, 14, 21, and 28 (20−μm sections; magnification: 100×; scale bars: 300 μm). (**B**) Quantification of immunofluorescence reactivity in the contralateral and ipsilateral SCDHs (4 sections per rat, three rats per group and each time point); values are expressed as means ± standard error of means (SEMs). * *p* < 0.05 relative to the same side of controls; # *p* < 0.05 relative to the contralateral side of the same group at the same time point, one-way analysis of variance (ANOVA) with post hoc Tukey test.

As shown in Figure 3, vWF immunoreactivity was significantly increased from POD 7 to 28 after CCI, with a peak at POD 14 in the ipsilateral spinal cord compared to that of controls (2.3-fold, *p* = 0.00125; 2.7-fold, *p* < 0.001; 2.4-fold, *p* < 0.001; and 1.7-fold, *p* < 0.001 at POD 7, 14, 21, and 28, respectively), while there was no difference on the contralateral side (1.1-, 1.2-, 1.2-, and 1.0-fold; all *p* > 0.05) (*n* = 3 per group). Furthermore, vWF immunoreactivity was also significantly upregulated in the ipsilateral SCDH after CCI at POD 7, 14, 21, and 28 (2.3- vs. 1.1-fold, *p* < 0.001; 2.7- vs. 1.2-fold, *p* < 0.001; 2.4- vs. 1.2-fold, *p* = 0.023; and 1.7- vs. 1.0-fold, *p* = 0.0141; respectively) compared to the contralateral side at the same time point (*n* = 3 per group; Figure 3). These data support the hypothesis that CCI activates angiogenesis in the ipsilateral lumbar spinal cord.

### 3.4. Neuroinflammation Enhances Pro-inflammatory Cytokine Release in the Ipsilateral Lumbar Spinal Cord after CCI

**Figure 4:**
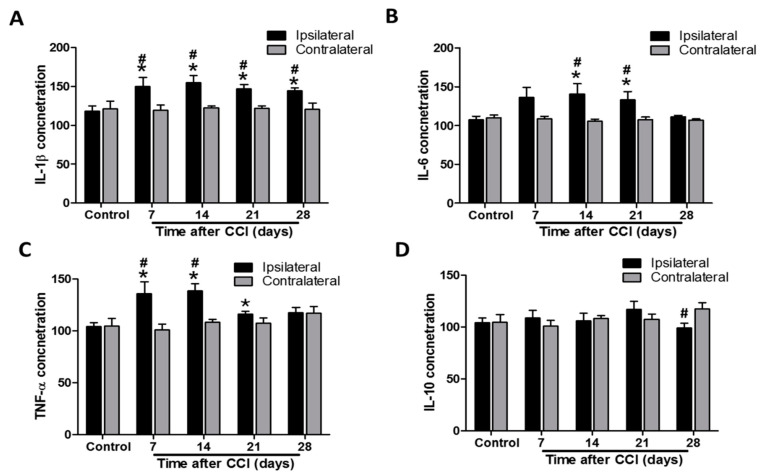
Time course assessment of cytokine levels in the lumbar spinal cord of control rats and chronic constriction injury (CCI) rats at post-operative day (POD) 7, 14, 21, and 28. The levels of interleukin-1β (IL-1β) (**A**), tumor necrosis factor-α (TNF-α) (**B**), IL-6 (**C**), and IL-10 (**D**) proteins were measured by immunoassay in the dorsal ipsilateral and contralateral parts of the lumbar enlargement. All values were expressed as pg/100 μg total proteins, and means ± standard error of means (SEMs) (*n* = 9 per group and each time point) are represented. * *p* < 0.05 when the protein levels in the same side of the dorsal part of the spinal cord at individual time points were compared to that in control rats. # *p* < 0.05 when the protein levels in the ipsilateral dorsal part of the spinal cord were compared to those in the contralateral side at the same timepoint, one-way analysis of variance (ANOVA) with post hoc Tukey test.

In control rats, IL-1β concentration in ipsilateral lumbar spinal cord dorsal part was not significantly different to that in the contralateral side (118.2 ± 6.9 vs. 121.2 ± 9.8 pg/100 μg proteins, *p* = 0.807; *n* = 9 at each side; Figure 4A). However, the IL-1β level was increased in the ipsilateral spinal cord at POD 7, 14, 21, and 28 after CCI compared to that in controls (149.9 ± 11.5, *p* = 0.0315; 154.8 ± 9.3, *p* = 0.00631; 146.8 ± 5.5, *p* = 0.00646; and 144.6 ± 3.3, *p* = 0.00493 respectively, vs. 118.2 ± 6.9 pg/100 μg proteins; *n* = 9 per group at each time point; Figure 4A). There were no differences in IL-1β concentration between the contralateral spinal cord of CCI rats and the controls (119.4 ± 6.8, 122.3 ± 2.3, 122.0 ± 3.0, and 120.6 ± 7.7 at POD 7, 14, 21, and 28, vs. 121.2 ± 9.8 pg/100 μg proteins, all *p* > 0.05; *n* = 9 per group at each time point; Figure 4A). Furthermore, the IL-1β level was significantly upregulated in the ipsilateral side after CCI at POD 7, 14, 21, and 28 (*p* = 0.037, 0.00384, 0.00102, and 0.0151, respectively) compared to the contralateral side at the same time point (Figure 4A). Similarly, IL-6 concentrations were not significantly different between ipsilateral and contralateral sides of lumbar spinal cord dorsal part in control rats (107.3 ± 4.6 vs. 110.1 ± 3.8 pg/100 μg proteins, *p* = 0.652; *n* = 9 per group at each side; Figure 4B). IL-6 levels were significantly elevated after CCI in the ipsilateral spinal cord at POD 7, 14, and 21 compared to that in controls (140.8 ± 13.4, *p* = 0.0292 and 133.1 ± 10.5, *p* = 0.0394, respectively, vs. controls 107.3 ± 4.6 pg/100 μg proteins) but not at POD 7 and 28 (136.2 ± 13.0, *p* = 0.0526 and 111.2 ± 2.1 pg/100 μg protein, *p* = 0.457, respectively) (*n* = 9 per group at each time point; Figure 4B). CCI had no effect on IL-6 concentration in the contralateral spinal cord at POD 7, 14, 21, and 28 compared to that of control rats (108.6 ± 3.3, 105.4 ± 3.0, 107.5 ± 3.5, and 106.8 ± 1.8, respectively, vs. 110.0 ± 3.8 pg/100 μg proteins, all *p* > 0.05; *n* = 9 per group at each time point; Figure 4B). Additionally, the IL-6 level was significantly upregulated in the ipsilateral side after CCI at POD 14 and 21 (*p* = 0.0187 and 0.0346, respectively), but not at POD 7 and 28 (*p* = 0.0567 and 0.136, respectively), compared to the contralateral side at the same time point (*n* = 9 per group at each time point; Figure 4B). TNF-α concentrations were also not significantly different between ipsilateral and contralateral sides of lumbar spinal cord dorsal part in control rats (104.0 ± 4.0 vs. 104.7 ± 7.3 pg/100 μg proteins, *p* = 0.941; *n* = 9 at each side; Figure 4C). TNF-α levels in the ipsilateral spinal cord were significantly elevated at POD 7, 14, and 21 in CCI rats (135.8 ± 11.8, *p* = 0.0294; 138.6 ± 6.9, *p* < 0.001, and 116.3 ± 2.5, *p* = 0.018, respectively), but not at POD 28 (117.6 ± 5.0 pg/100 μg proteins, *p* = 0.0563), compared to that in controls (104.0 ± 4.0 pg/100 μg proteins; *n* = 9 per group at each time point; Figure 4C). Similarly, TNF-α concentrations were not affected in the contralateral side after CCI at POD 7, 14, 21, and 28 (100.9 ± 5.6, 108.6 ± 5.6, 107.3 ± 5.0, and 117.3 ± 6.3, respectively, vs. controls 104.7 ± 7.3 pg/100 μg proteins, all *p* > 0.05; *n* = 9 per group at each time point; Figure 4C). Furthermore, the TNF-α level was significantly upregulated in the ipsilateral side after CCI at POD 7 and 14 (*p* = 0.0173 and < 0.001, respectively), but not at POD 21 and 28 (*p* = 0.128 and 0.967 respectively), compared to the contralateral side at the same time point (*n* = 9 per group at each time point; Figure 4C).

IL-10 concentrations were also not significantly different between ipsilateral and contralateral sides of the lumbar spinal cord dorsal part in control rats (104.2 ± 4.5 vs. 104.7 ± 7.3 pg/100 μg proteins, *p* = 0.956; *n* = 9 at each side; Figure 4D). IL-10 levels in the lumbar spinal cord dorsal part were unaffected by CCI at POD 7, 14, 21, and 28 compared to the controls (for the ipsilateral side, 108.9 ± 7.4, *p* = 0.596; 106.2 ± 7.4, *p* = 0.823; 116.9 ± 8.0, *p* = 0.185; and 98.9 ± 4.9, *p* = 0.441, respectively, vs. 104.2 ± 4.5 pg/100 μg proteins; for the contralateral side, 100.9 ± 5.6, 108.6 ± 2.7, 107.3 ± 5.0, and 117.3 ± 6.3, respectively, vs. 104.7 ± 7.3 pg/100 μg proteins; all *p* > 0.05; *n* = 9 per group at each time point; Figure 4D). However, the IL-10 level was significantly decreased in the ipsilateral side after CCI at POD 28 (*p* = 0.0359), but not at POD 7, 14, and 21 (*p* = 0.402, 0.766, and 0.324 respectively), compared to the contralateral side at the same time point (*n* = 9 per group at each time point; Figure 4D). These data demonstrate that CCI induces a pro-inflammatory cytokine response in the ipsilateral lumbar spinal cord.

### 3.5. Intrathecal Administration of Fumagillin and Anti-VEGF-A Monoclonal Antibodies Attenuates CCI-Induced Neuropathic Pain

**Figure 5:**
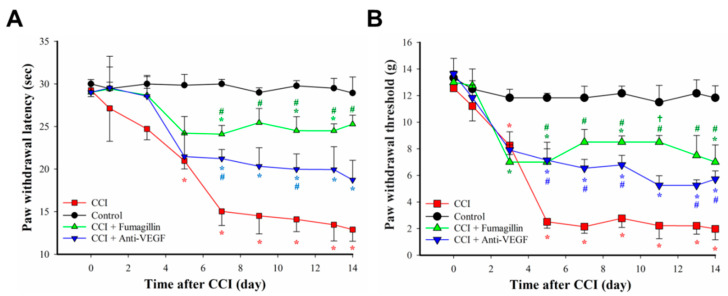
Effects of intrathecal administration of anti−vascular endothelial growth factor−A (anti−VEGF−A) monoclonal antibodies (0.3 μg/day) and fumagillin (0.1 μg/day) for 14 consecutive days on chronic constriction injury (CCI)−induced nociceptive behaviors. Thermal hyperalgesia, assessed by paw withdrawal latency (PWL) measurement (**A**), and mechanical allodynia, assessed by paw withdrawal threshold (PWT) measurement (**B**), were induced by CCI in rats. Data are expressed as means ± standard error of means (SEMs) of PWL in seconds and PWT in grams (*n* = 3 rats for control, CCI + fumagillin, and CCI + anti-VEGF groups; *n* = 5 for CCI group). * *p* < 0.05 relative to the control group; # *p* < 0.05 relative to the CCI group; † *p* < 0.05 relative to the CCI + anti-VEGF group; one-way analysis of variance (ANOVA) with post hoc Tukey test.

The dose–response effect of fumagillin on CCI-induced pain behavior is shown in the supplementary materials (*n* = 3 per group and each time point; Figure S3). Fumagillin had no analgesic effect on naïve and sham-operated rats for a 0.01–1 μg dose range. However, a trend toward decreased neuropathic pain in CCI rats was observed within 3 h after a single intrathecal injection of 0.1 and 1 μg fumagillin. Intrathecal administration of an anti-VEGF-A antibody, at a dose of 0.3 μg/day for 14 consecutive days, reduced the prolonged time to cross the beam and changed the hindlimb weight distribution induced by CCI (*n* = 3 in control group, *n* = 3 in CCI group, and *n* = 4 in CCI + anti-VEGF group; Figure S4 in supplementary materials). Therefore, we used intrathecal administration of fumagillin (0.1 μg/day) or anti-VEGF-A antibodies (0.3 μg/day) once a day for 14 consecutive days after CCI to examine the role of angiogenesis in CCI-induced neuropathic pain. The baseline nociceptive response to radiant heat and to a mechanical stimulus was comparable in all groups (*p* > 0.05; *n* = 3 for control, CCI + fumagillin, and CCI + anti-VEGF groups; *n* = 5 for CCI group; Figure 5). Time course studies showed a marked time-dependent reduction of the PWL in response to radiant heat (21.0 ± 1.0 vs. 29.9 ± 1.3 s, *p* = 0.008; 15.0 ± 1.7 vs. 30.0 ± 0.5 s, *p* < 0.001; 14.5 ± 2.1 vs. 29.0 ± 0.5 s, *p* < 0.001; 14.1 ± 1.4 vs. 29.8 ± 0.6 s, *p* < 0.001, 13.5 ± 1.9 vs. 29.5 ± 1.2 s, *p* < 0.001, and 12.9 ± 1.4 vs. 28.9 ± 1.9 s, *p* < 0.001, at POD 5, 7, 9, 11, 13, and 14, respectively; *n* = 3 for control, CCI + fumagillin, and CCI + anti-VEGF groups; *n* = 5 for CCI group; Figure 5A) from POD 5 to 14 and PWT triggered by a mechanical stimulus (8.2 ± 0.7 vs. 11.8 ± 0.6, *p* = 0.0127 at POD 3; 2.5 ± 0.5 vs. 11.8 ± 0.3, 2.1 ± 0.5 vs. 11.8 ± 0.9, 2.8 ± 0.7 vs. 12.2 ± 0. 5, 2.2 ± 1.0 vs. 11.5 ± 1.3, 2.2 ± 0.6 vs. 12.2 ± 1.0, and 2.0 ± 0.8 vs. 11.8 ± 0.9 g at POD 5, 7, 9, 11, 13, and 14, respectively; all *p* < 0.001; *n* = 3 for control, CCI + fumagillin, and CCI + anti-VEGF groups; *n* = 5 for CCI group; Figure 5B) from POD 3 to 14 for the ipsilateral hindpaw of the CCI group compared to control rats. These data indicate that CCI progressively induces thermal hyperalgesia and mechanical allodynia within the 14-day postoperative observation period, with the most remarkable effect at POD 14. Fumagillin significantly ameliorated the reduced PWL induced by CCI from POD 7 to 14 (24.1 ± 1.0 vs. 15.0 ± 1.7 s, *p* = 0.002; 25.5 ± 1.7 vs. 14.5 ± 2.1 s, *p* = 0.006; 24.5 ± 1.6 vs. 14.1 ± 1.4 s, *p* = 0.001; 24.5 ± 0.8 vs. 13.5 ± 1.9 s, *p* = 0.005; and 25.3 ± 1.0 vs. 12.9 ± 1.4 s, *p* < 0.001 at POD 7, 9, 11, 13, and 14, respectively) but the anti-VEGF-A antibody only significantly improved it at POD 7 and POD 11 (21.2 ± 1.1 vs. 15.0 ± 1.7 s, *p* = 0.026 and 20.0 ± 1.8 vs. 14.1 ± 1.4 s, *p* = 0.045) (Figure 5A). Compared to the PWL of control rats, the PWL was significantly reduced in the CCI + fumagillin group at POD 7 (24.1 ± 1.0 vs. 30.0 ± 0.5 s, *p* = 0.007) and in the CCI + anti-VEGF group at POD 7, 9, 11, 13, and 14 (21.2 ± 1.1 vs. 30.0 ± 0.5 s, *p* < 0.001; 21.2 ± 1.1 vs. 29.0 ± 0.5 s, *p* = 0.016; 20.0 ± 1.8 vs. 29.8 ± 0.6 s, *p* = 0.006; 19.9 ± 2.7 vs. 29.5 ± 1.2 s, *p* = 0.015; and 18.7 ± 2.3 vs. 28.9 ± 1.9 s, *p* = 0.013, respectively; Figure 5A). These results suggest that fumagillin is more efficient than the anti-VEGF-A antibody in suppressing CCI-induced thermal hyperalgesia although the difference was not statistically significant (all *p* > 0.05; Figure 5A).

Similarly, fumagillin significantly ameliorated the PWT from POD 5 to 14 (7.0 ± 1.0 vs. 2.5 ± 0.5 g, *p* = 0.006; 8.5 ± 1.0 vs. 2.1 ± 0.5 g, *p* < 0.001; 8.5 ± 0.5 vs. 2.8 ± 0.7 g, *p* < 0.001; 8.5 ± 0.5 vs. 2.2 ± 1.0 g, *p* = 0.002; 7.5 ± 1.5 vs. 2.2 ± 0.6 g, *p* = 0.004; 7.0 ± 1.3 vs. 2.0 ± 0.8 g, *p* = 0.008, at POD 5, 7, 9, 11, 13, and 14, respectively) but the anti-VEGF-A antibody only significantly improved it at POD 5 (7.1 ± 1.4 vs. 2.5 ± 0.5 g, *p* = 0.005), POD 7 (6.5 ± 0.7 vs. 2.1 ± 0.5 g, *p* = 0.003), POD 9 (6.8 ± 0.7 vs. 2.8 ± 0.7 g, *p* = 0.004), and POD 14 (5.7 ± 0.6 vs. 2.0 ± 0.8 g, *p* = 0.04) compared to the PWT of CCI rats (Figure 5B). Compared to that of controls, the PWT was significantly decreased in the CCI + fumagillin group at POD 5, 9, 13, and 14 (7.0 ± 1.0 vs. 11.8 ± 0.3 g, *p* = 0.024; 8.5 ± 0.5 vs. 12.2 ± 0. 5 g, *p* = 0.001; 7.50 ± 1.5 vs. 12.2 ± 1.0 g, *p* = 0.039; and 7.0 ± 1.3 vs. 11.8 ± 0.9 g, *p* = 0.023, respectively; Figure 5B) and in the CCI + anti-VEGF group from POD 5 to 14 (7.1 ± 1.4 vs. 11.8 ± 0.3 g, *p* = 0.027; 6.5 ± 0.7 vs. 11.8 ± 0.9 g, *p* = 0.008; 6.8 ± 0.7 vs. 12.2 ± 0.5 g, *p* = 0.008; 5.3 ± 0.7 vs. 11.5 ± 1.3 g, *p* = 0.005; 5.3 ± 0.4 vs. 12.2 ± 1.0 g, *p* = 0.007, and 5.7 ± 0.6 vs. 11.8 ± 0.9 g, *p* = 0.008, at POD 5, 7, 9, 11, 13, and 14, respectively; Figure 5B). Fumagillin showed a tendency to be more efficient than the anti-VEGF-A antibody in suppressing the CCI-induced mechanical allodynia although the differences were only statistically significant at POD 11 (8.5 ± 0.5 vs. 5.3 ± 0.7 g, *p* = 0.0207; Figure 5B).

In summary, intrathecal administration of fumagillin (0.01–0.1 μg) and anti-VEGF-A antibodies (0.3 μg) did not affect the behavior of control rats but improved CCI-induced pain behaviors. Because of its stronger effect on attenuating CCI-induced nociceptive behaviors, fumagillin (0.1 μg/day) was used in the following experiments to evaluate its effect on CCI-induced angiogenesis, astrocyte activation, and dysregulated pro/anti-inflammatory cytokine balance in the spinal cord.

### 3.6. Intrathecal Fumagillin Attenuates CCI-Induced Angiogenesis in the Lumbar Spinal Cord

**Figure 6:**
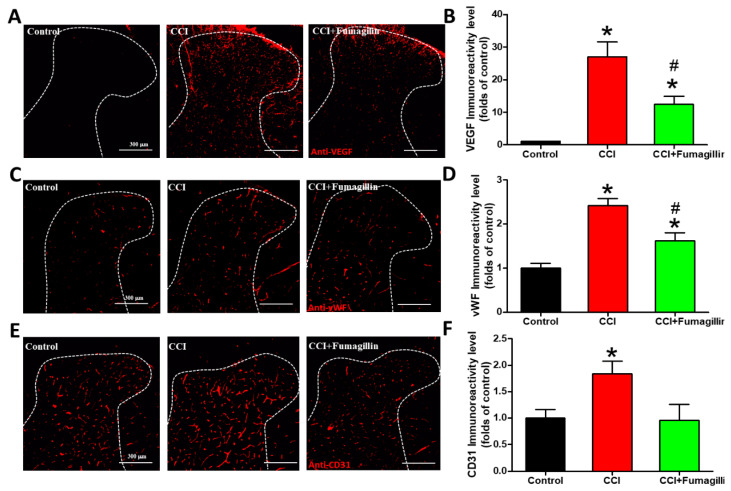
Effect of intrathecal administration of fumagillin on chronic constriction injury (CCI)−induced angiogenesis. Lumbar spinal cords were obtained at postoperative day (POD) 14 after the last intrathecal injection from control, CCI, and CCI + fumagillin groups. Immunofluorescence images show cells labeled with vascular endothelial growth factor (VEGF) (red; **A**), von Willebrand factor (vWF) (red; **C**), and cluster of differentiation 31 (CD31) (red; **E**) in the ipsilateral spinal cord dorsal horn (SCDH) (20−μm sections; magnification: 100×; scale bars: 300 μm). Quantification of VEGF (**B**), vWF (**D**), and CD31 (**F**) immunoreactivities are shown as means ± standard error of means (SEMs) (4 sections per rat, 3 rats per group). * *p* < 0.05 compared with the control group; # *p* < 0.05 compared with the CCI group; one−way analysis of variance (ANOVA) with post hoc Tukey test.

The effects of anti-angiogenic therapy on CCI-produced central sensitization were assessed at POD 14 since CCI-induced the most significant angiogenesis at this time point. Fumagillin significantly decreased the CCI-induced upregulation of VEGF and vWF immunoreactivities (12.4- vs. 27.0-fold, *p* = 0.0486 and 1.6- vs. 2.4-fold, *p* = 0.0266, respectively; Figure 6A–D), but not CD31 immunoreactivities (1.0- vs. 1.8-fold, *p* = 0.0841; Figure 6E,F), in ipsilateral lumbar SCDH (*n* = 3 per group). The extent of fumagillin-reduced expression of the angiogenic factor was strongest on VEGF (−54%), followed by CD31 (−48%) and vWF (−33%), whereas no significant difference was found in CD31 expression (Figure 6B,D,F). However, VEGF and vWF immunoreactivities in the CCI + fumagillin group were still more intense than that of the control group (12.4- vs. 1-fold, *p* = 0.01, and 1.6- vs. 1-fold, *p* = 0.0409, respectively), while there was no difference in CD31 staining (1.0- vs. 1.0-fold, *p* = 0.907) (*n* = 3 per group; Figure 6B,D,F). These data demonstrate that fumagillin effectively improves CCI -induced angiogenesis in the ipsilateral lumbar spinal cord.

### 3.7. Fumagillin Differentially Modulates CCI-Induced Spinal Cytokine Production

**Figure 7:**
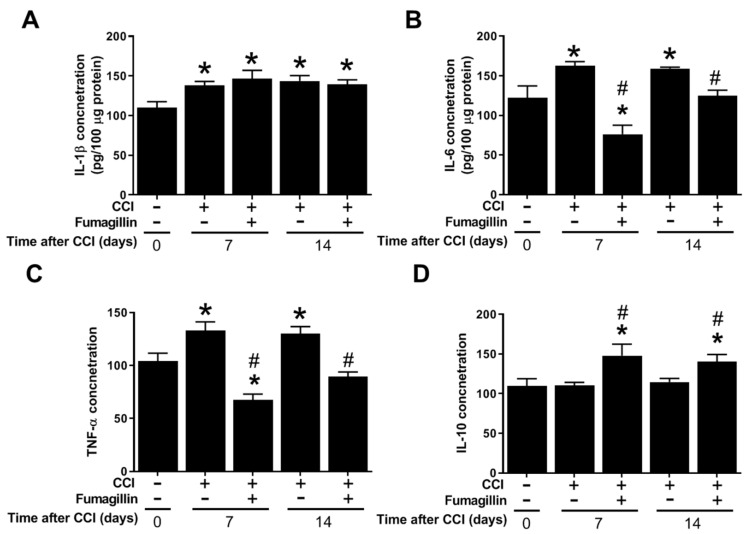
Effects of intrathecal administration of fumagillin on cytokine levels in the spinal cord of CCI rats 7 and 14 days following CCI. (**A**) interleukin−1β (IL−1β), (**B**) tumor necrosis factor−α (TNF−α), (**C**) IL−6, and (**D**) IL−10 levels in the spinal cord of control rats (*n* = 8) and chronic constriction injury (CCI) rats at post−operative day (POD) 7 (*n* = 8), CCI + fumagillin rats at POD 7 (*n* = 6), CCI rats at POD 14 (*n* = 6), and CCI + fumagillin rats at POD 14 (*n* = 8). Data are represented as means ± standard error of means (SEMs), * *p* < 0.05 compared with the control group; # *p* < 0.05 compared with the CCI group at POD 7; one-way analysis of variance (ANOVA) with post hoc Tukey test. The “−” sign means the absence and “+” sign means presence of various treatment with either CCI or fumagillin in the rats..

IL-1β levels did not significantly change at POD 7 (*n* = 6) and 14 (*n* = 8) in the CCI + fumagillin group compared to that of the CCI group (*n* = 8 at POD 7 and *n* = 6 at POD 14) (146.7 ± 11.9 vs. 138.0 ± 7.1, *p* = 0.486, and 139.3 ± 7.23 vs. 143.5 ± 7.9 pg/100 μg proteins, *p* = 0.652; Figure 7A). However, fumagillin dramatically reduced the amounts of IL-6 (76.2 ± 16.2 vs. 162.7 ± 7.4 and 124.7 ± 10.3 vs. 159.1 ± 2.0 pg/100 μg proteins at POD 7 and 14, respectively, both *p* < 0.001; Figure 7B) and TNF-α (67.5 ± 7.9 vs. 133.2 ± 10.8 and 89.5 ± 5.0 vs. 130.2 ± 9.2 pg/100 μg proteins at POD 7 and 14, respectively, both *p* < 0.001; Figure 7C) at POD 7 and 14 compared to that of the CCI group (*n* = 8 rats for CCI group at POD 7 and CCI + fumagillin group at POD 14; *n* = 6 for CCI group at POD 14 and CCI + fumagillin group at POD 7). Furthermore, the effect of fumagillin in suppressing CCI-upregulated TNF-α and IL-6 concentrations at POD 7 tended to be stronger than that at POD 14, albeit not statistically significant (Figure 7B,C). Unexpectedly, fumagillin significantly increased IL-10 levels at POD 7 and 14 compared to that of CCI rats (147.9 ± 20.6 vs. 110.3 ± 4.8, *p* = 0.0076, and 140.4 ± 12.7 vs. 114.6 ± 5.2 pg/100 μg proteins, *p* = 0.0332). The increased IL-10 production induced by fumagillin seemed greater at POD 7 than that at POD 14; however, the difference was not statistically significant (Figure 7D). In summary, fumagillin modulates CCI-induced neuroinflammation by enhancing IL-10 amounts and inhibiting the production of TNF-α and IL-6 without affecting IL-1β levels.

### 3.8. Fumagillin Intrathecal Administration Attenuates CCI-Induced Astrocyte Activation in the Ipsilateral Lumbar Spinal Cord

**Figure 8:**
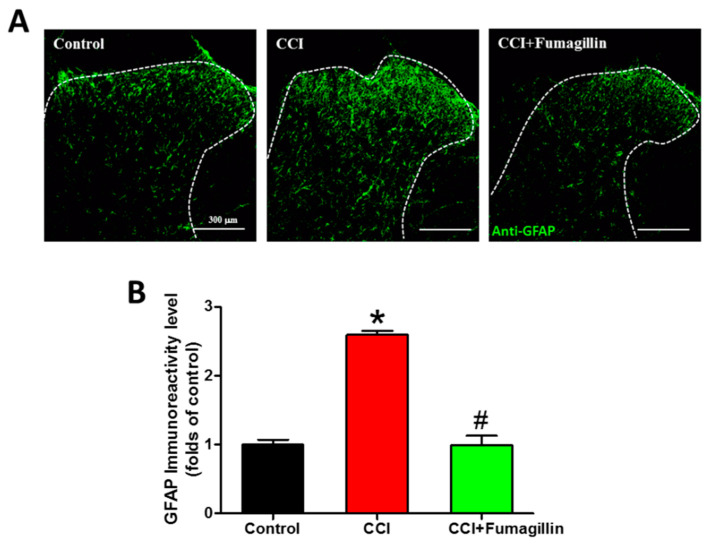
Effect of intrathecal administration of fumagillin on chronic constriction injury (CCI)−induced astrocytic activation. Lumbar spinal cord sections were obtained at postoperative day (POD) 14 from control, CCI, and CCI + fumagillin groups. (**A**) Immunofluorescence images show cells labeled with glial fibrillary acidic protein (GFAP, green) in the ipsilateral spinal cord dorsal horn (20−μm sections; magnification: 100× scale bars: 300 μm). (**B**) The quantification of GFAP immunoreactivity was expressed as mean ± standard error of mean (SEM) (4 sections per rat, 3 rats per group). Treatment with fumagillin (intrathecal) significantly inhibited CCI−upregulated GFAP immunoreactivity. * *p* < 0.05 compared with the control group; # *p* < 0.05 compared with the CCI group; one-way analysis of variance (ANOVA) with post hoc Tukey test.

CCI markedly upregulated GFAP immunoreactivities at POD 14 compared to that of the control group (1.7- vs. 1-fold, *p* < 0.001; Figure 8). GFAP expression was decreased in the CCI + fumagillin group compared to that of the CCI group (0.6- vs. 1.7-fold, *p* = 0.00278) but not statistically significant compared to that of control groups (0.6- vs. 1-fold, *p* = 0.232) (*n* = 3 per group; Figure 8A,B). These data demonstrate that intrathecal administration of fumagillin effectively abolishes astrocyte activation in the ipsilateral lumbar spinal cord in CCI-induced neuropathic pain.

## 4. Discussion

The present study demonstrates that CCI generates neuropathic pain in rats, induces astrocyte activation and an upregulated expression of angiogenic factors, and enhances pro-inflammatory cytokines in the ipsilateral lumbar spinal cord. Once-daily intrathecal administration of fumagillin and anti-VEGF-A antibody for 14 days immediately after CCI significantly reduced the severity of CCI-induced neuropathic pain. In addition, fumagillin attenuated CCI-induced angiogenesis and astrocyte activation in the spinal cord. Fumagillin also inhibited CCI-induced neuroinflammation in the spinal cord by differentially modulating cytokine production and changing the balance between pro- and anti-inflammatory cytokines. These results indicate that CCI induces angiogenesis in the spinal cord, which participates in astrocyte activation, neuroinflammation, and neuropathic pain.

CCI of the sciatic nerve in rodents is a widely used animal model of peripheral neuropathic pain. Neurons and glial cells participate in developing and maintaining nociceptive sensitization in CCI models [7,31,33,38]. Several lines of evidence suggest that elevated energy consumption, high metabolism, and increased blood flow in the CNS are generated by neuronal–glial interactions and neuroinflammation in pain. Pain increases regional cerebral blood flow and metabolism in the somatosensory cortex, limbic system, and brainstem of the resting, unstimulated brain from 12–14 days to 12 weeks, as evidenced by micro-positron emission tomography with a [18F]fluorodeoxyglucose imaging study or densitometric analysis of 99mTc autoradiograms of brain sections from rat models of peripheral neuropathic pain [23,24,39]. Holland-Fischer et al. demonstrated by indirect calorimeter and plasma catecholamine and urine urea nitrogen assessments that an acute increase in energy expenditure and glucose oxidation occurs during non-traumatic, painful electrical stimulation of human abdominal skin and is abolished by local analgesia [40]. The authors suggested that the energy expenditure increase was mediated by elevated adrenergic activity, increased muscle tone, and pain-related cortical events and emphasized the importance of pain management to reduce pain-evoked adverse effects [40]. Single-photon emission computed tomography revealed a significant cerebral blood flow increase in the right anterior cingulate cortex of patients with diabetic neuropathic pain [41]. Transcranial doppler sonography showed increased cerebral blood flow in anterior and middle cerebral arteries during a painful stimulus to the index fingernail in fibromyalgia patients [15]. Additionally, the upregulated expression of VEGF signaling molecules (VEGF mRNA for POD 7–21, VEGF receptor 2 (VEGFR 2) mRNA for POD 3–21, and both proteins for POD 5–21) in the activated neurons and microglia in the ipsilateral lumbar SCDH closely participated in the pain behavior (thermal hyperalgesia from POD 7 to 21 and mechanical allodynia from POD 5 to 21) in a female rat model of metastatic breast cancer bone pain. Intrathecal post-treatment with inhibitors of VEGF signaling significantly attenuated cancer pain behaviors [25]. However, evidence that angiogenesis in the CNS directly supports long-term activation of neurons and glia in chronic pain is lacking. Here, we assessed the levels of three different angiogenic factors, VEGF, CD31, and vWF, to identify the role of angiogenesis in central nociceptive sensitization in the spinal cord induced by peripheral neuropathic insults.

Angiogenic factors play an important role in forming new blood vessels from the existing vasculatures [42]. In particular, vWF, a multimeric glycoprotein localized in endothelial cells and megakaryocytes, is bound to endothelial and vascular smooth muscle cells [43] and is considered a marker of endothelial cells. It has many roles in the vasculature, including hemostasis, the control of inflammation by modulating leukocyte adhesion and migration, the regulation of vascular permeability, and angiogenesis [43,44,45]. CD31 is a glycosylated transmembrane adhesion protein highly expressed on endothelial cells and hematopoietic-derived cells, including macrophages, lymphocytes, and platelets. CD31 plays an important role in angiogenesis in maintaining vascular permeability, leukocyte migration and inflammation, and platelet-endothelial cell adhesion regulation [46,47]. VEGF, a major angiogenic factor acting on endothelial cells to increase blood vessel density, has been found upregulated in the spinal cord of multiple sclerosis patients and in rat models of EAE and spinal cord contusion injury [19,48]. VEGF, CD31, and vWF are recognized endothelial cell markers used to evaluate the presence of endothelial cells, vascular density, and angiogenesis. Our findings demonstrate that CCI significantly and simultaneously upregulates the expression of these angiogenic factors in the ipsilateral lumbar spinal cord from POD 7 to 28 with a peak at POD 14. This is consistent with the upregulated VEGF mRNA expression observed after bone cancer inoculation in female rats [25]. To our knowledge, the time course of angiogenesis, e.g., CD31 and vWF expressions, has not been examined in chronic pain, and our results provide evidence that CCI induces angiogenesis in the spinal cord.

Angiogenesis in the osteochondral junction, synovium, and meniscus participate in the pathological processes of human osteoarthritis. An association between angiogenesis, subchondral inflammation, synovitis, and the extension of unmyelinated sensory nerves accompanying blood vessels in the osteochondral junction appears in patients with osteoarthritis and rheumatoid arthritis [49]. The present study reveals an upregulated expression of angiogenic factors in the rat spinal cord after CCI. We propose that this increased expression results in new vessel formation to provide more blood, oxygen, and nutrients to support central sensitization, neuronal–glial interaction, and neuroinflammation. It constitutes a pathophysiological mechanism of chronic pain since anti-angiogenic treatment attenuates pain. Indeed, anti-angiogenic therapy with PPI-2458 (fumagillin analog) reduces synovial and osteochondral angiogenesis, synovial inflammation, joint damage, and pain behavior in a rat model of meniscal-transection-induced osteoarthritis. Bevacizumab (anti-VEGF-A monoclonal antibody) reduces osteoarthritis severity and weight-bearing pain in a rabbit model of osteoarthritis induced by anterior cruciate ligament transection [50,51]. Therefore, angiogenesis, including elevated VEGF levels, might enhance inflammation, structural damages, and pain in osteoarthritis. It might be treated by anti-angiogenic therapy [52,53]. Angiogenesis is also critical for tumor growth and metastasis. VEGF is a key mediator of tumor angiogenesis. VEGF also participates in pain sensitization in the spinal cord and dorsal root ganglia (DRG) [25,26]. Exogenous VEGF perfusion increased the spontaneous excitatory postsynaptic currents in lamina II spinal neurons in a whole-cell patch-clamp study, and intrathecal VEGF administration produced time-dependent nociceptive pain within 1 h, persisting for at least 12 h in naïve rats [25]. Upregulation of VEGF and VEGFR 2 expression occurred predominately in the ipsilateral, but not contralateral, SCDH after cancer inoculation. Intrathecal administration of VEGF neutralizing antibodies or VEGFR 2 inhibitors significantly attenuated cancer bone pain in rat models [25]. Similarly, VEGF and VEGFR 2 expression were elevated in L4–6 DRG in CCI rats. Intrathecal injection of the anti-VEGF antibody dramatically abolished the CCI-induced neuropathic pain behaviors and upregulation of VEGF and VEGFR 2 expression [26]. Therefore, angiogenesis and VEGF/VEGFR 2 signaling might have a role in peripheral and central sensitizations. The present study demonstrates that CCI simultaneously upregulates VEGF, CD31, and vWF expression in a time-dependent manner in the ipsilateral lumbar spinal cord between POD 7 and 28. This increased expression occurs during the development and maintenance of CCI-induced nociceptive pain. This agrees with Hu et al., who reported [25] that anti-angiogenic therapy with fumagillin or anti-VEGF-A antibodies during a 14-day observation period improves CCI-induced thermal hyperalgesia between POD 7 and 14 and mechanical allodynia between POD 5 and 14, which closely relates to the time course of CCI-induced pain behavior. Angiogenesis in the ipsilateral spinal cord plays an important role in developing and maintaining chronic neuropathic pain.

Astrocytes have versatile functions in the trophic support of the neurovascular unit by wiring neurons to vessels to maintain the physiological functions of the CNS, synaptic transmission, microenvironmental regulation, neuroinflammation, and pain regulation [13,30,54]. Astrocytes regulate angiogenesis by various mechanisms, including modulation of VEGF and hypoxia-inducible factor α-Wnt/β-catenin signaling [55,56]. Astrocyte activation, neuroinflammation with increased production of inflammatory mediators, and neurovascular endothelial activation with increased CD31 expression occurred in the thoracic spinal cord in a rat model of experimental pulmonary hypertension [57]. CCI induced the activation of spinal astrocytes and increased the production of pro-inflammatory mediators (TNF-α, IL-1β, and IL-6) in the male rat lumbar spinal cord [58,59,60,61]. In contrast, inhibiting astrocytes prevents the release of inflammatory cytokines and neuropathic pain in CCI rats [58]. Jančálek et al. found elevated TNF-α and IL-10 levels bilaterally in cervical and lumbar DRG of female rats, following CCI of the left sciatic nerve. TNF-α amounts increased from POD 7 to 14, while IL-10 levels increased in lumbar DRG between POD 1 and 3 and decreased in cervical and lumbar DRGs from POD 7 to reach normalized values at POD 14 [62]. Our previous studies showed an upregulation of TNF-α expression in spinal microglia and astrocytes after CCI. Intrathecal administration of lemnalol significantly inhibited CCI-induced TNF-α expression in astrocytes and microglia [31]. Intrathecal injection of adenoviral-mediated transfer of phosphatase and tensin homolog (Ad-PTEN) inhibited it primarily in astrocytes [7]. However, Ji et al. suggested that activated astrocytes produce IL-1β. At the same time, activated microglia synthesize both TNF-α and IL-1β in the spinal cord to participate in neuroinflammation, central sensitization, and pain hypersensitivity in chronic pain [13]. Additionally, Hu et al. found that pro-inflammatory cytokine mRNA expression (TNF-α, IL-1β, IL-6, and IL-18) is upregulated in the rat SCDH after cancer inoculation, and pharmacological inhibition of VEGF-A signaling effectively reduces this tumor-induced mRNA expression in spinal microglia [25]. Furthermore, pro-inflammatory cytokines, including TNF-α and IL-1β, were shown to contribute to angiogenesis [63,64]. Owing to the high energy expenditure related to the pathophysiology of neuroinflammation and the divergent results regarding the major source of cytokine production in the CNS in chronic pain, we speculated that anti-angiogenic therapy would inhibit CCI-induced astrocytic activation and, consequently, suppress pro-inflammatory cytokine production. The present study showed that, during a 28-day observation period, CCI generated neuroinflammation and an elevated production of the pro-inflammatory cytokines IL-1β from POD 7 to 28, IL-6 from POD 14 to 21, and TNFα from POD 7 to 21 in the ipsilateral lumbar spinal cord. The increased levels persisted for the longest for IL-1β, for an intermediary time for TNF-α, and for the shortest time for IL-6. Therefore, we propose that TNF-α and IL-6 play a role in developing CCI-induced neuroinflammation, while IL-1β is involved in its development and maintenance. CCI did not affect the anti-inflammatory cytokine IL-10. Fumagillin markedly abolished the CCI-induced increase of TNF-α and IL-6 production but did not affect IL-1β production. Interestingly, it significantly increased IL-10 synthesis in the ipsilateral spinal cord. Therefore, intrathecal administration of fumagillin for 14 days displaced the dysregulation of cytokine homeostasis from the pro-inflammatory signaling induced by CCI to an anti-inflammatory context by increasing IL-10 and reducing TNF-α and IL-6 production. Astrocyte immunoreactivity increased in the ipsilateral SCDH after CCI; fumagillin dramatically suppressed it. These findings suggest that astrocytes are a major source of CCI-induced pro-inflammatory cytokines, especially TNF-α and IL-6, in the ipsilateral spinal cord. Additionally, the fumagillin anti-inflammatory effect depends largely on inhibiting astrocyte activation and TNF-α and IL-6 production and increasing IL-10 synthesis.

**Figure 9:**
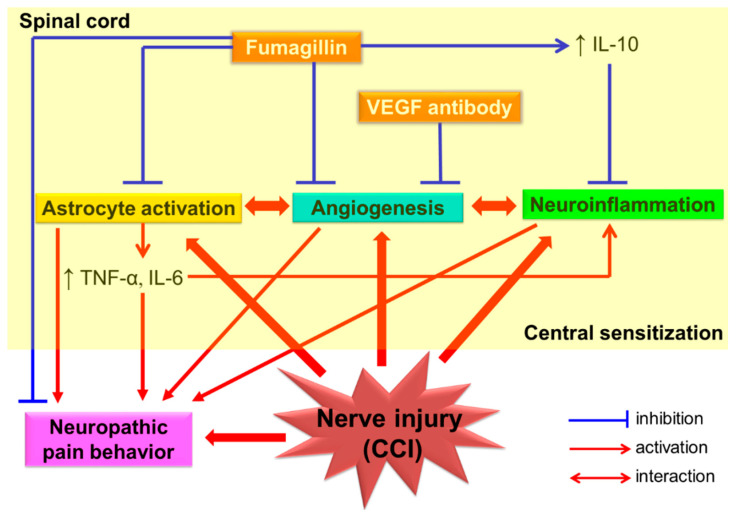
Schematic representation of the proposed mechanism for the anti-nociceptive effect of intrathecal fumagillin on spinal neuroinflammation and central sensitization in a rat model of chronic constriction injury (CCI)−induced neuropathic pain. Abbreviation: CCI, chronic constriction injury; IL, interleukin; TNF−α, tumor necrosis factor−α; ↑, upregulation.

Our results indicate that fumagillin is a versatile agent, suppressing CCI-induced upregulation of the expression of three angiogenic factors (VEGF, CD31, and vWF), regulating CCI-induced activation of spinal glia and cytokine production, and inhibiting CCI-induced neuropathic pain. We suggest that intrathecal post-treatment with fumagillin once a day for 14 consecutive days is an effective pharmacological intervention to inhibit CCI-induced neuroinflammation, astrocyte activation, pro-inflammatory cytokines TNF-α and IL-6 production, and angiogenesis in the spinal cord and to inhibit neuropathic pain in rats. It also has an anti-inflammatory/neuroprotective effect against CCI-induced central sensitization by increasing IL-10 production. Moreover, the present study provides evidence that astrocytes are the major cellular source of the TNF-α and IL-6 produced in response to CCI in rats (Figure 9).

The precise mechanisms involved in fumagillin-generated anti-angiogenic and anti-inflammation effects on central sensitization in CCI-induced neuropathic pain require further investigation. The present study has some limitations. (1) We measured the expression of angiogenic factors and not regional spinal blood flow or blood vessel density in the spinal cord directly. (2) We did not examine the cellular and molecular mechanisms of angiogenesis-related to neuroinflammation. (3) We did not investigate the role of microglia in fumagillin-inhibited neuroinflammation. However, microglia play an important role in central nociceptive sensitization. Microglia could be the source of IL-1β because fumagillin suppressed the activation of astrocytes after CCI but did not alter IL-1β levels. (4) We did not examine the cellular source of IL-10 increased production induced by fumagillin. Further studies are needed to determine whether the adverse events associated with anti-angiogenic treatments can be avoided.

## 5. Conclusions

In conclusion, fumagillin significantly prevents upregulated angiogenic factor expression and astrocyte activation and modulates the production of pro-inflammatory/anti-inflammatory cytokines in the ipsilateral spinal cord. It also alleviates the neuropathic pain induced by CCI. Our findings provide evidence that angiogenesis in the spinal cord plays an important role in developing CCI-induced neuropathic pain and central sensitization. Therefore, anti-angiogenic therapy might be a potential pharmacologic intervention for neuropathic pain.

## Data Availability

The data that support the findings of this study are available from the corresponding author upon reasonable request (sung6119@gmail.com).

## Supplementary Materials

The following are available online at https://www.mdpi.com/article/10.3390/biomedicines9091187/s1, Figure S1: Experimental design. Figure S2: Representative uncropped images of Western Blots in Figure 1C: VEGF and β-actin. Figure S3: Effects of fumagillin intrathecal administration on the pain behaviors evoked by chronic constriction injury (CCI) in rats. Figure S4: Effects of intrathecal administration of anti-vascular endothelial growth factor−A (anti−VEGF−A) monoclonal antibodies (0.3 μg/day) for 14 consecutive days on chronic constriction injury (CCI)-induced nociceptive behaviors.

## Abbreviations

aCSF: Artificial cerebrospinal fluid; Ad-PTEN: Adenoviral-mediated transfer of phosphatase and tensin homolog; CCI: Chronic constriction injury; CD31: Cluster of differentiation 31; CNS: Central nervous system; DRG: Dorsal root ganglia; EAE: Experimental autoimmune encephalomyelitis; GFAP: Glial fibrillary acidic protein antibody; IL: Interleukin; i.t.: Intrathecal; MetAP2: Methionine aminopeptidase type 2; MPE: Maximal possible effect; NF: Neurofibromatosis; PBS: Phosphate-buffered saline; POD: Postoperative day; PWL: Paw withdrawal latency; PWT: Paw withdrawal threshold; SCDH: Dorsal horn of the spinal cord; SEM: Standard error of mean; 99 mTc: Technetium-99m; TNF: Tumor necrosis factor; VEGF: Vascular endothelial growth factor; VEGFR: VEGF receptor; vWF: von Willebrand factor.

## References

[1] Willey J.Z., Grotta J.C., Albers G.W., Broderick J.P., Kasner S.E., Lo E.H., Mendelow A.D., Sacco R.L., Wong L.K.S. (2016). Stroke and Other Vascular Syndromes of the Spinal Cord. Stroke, Pathophysiology, Diagnosis, and Management.

[2] Biswas S., Cottarelli A., Agalliu D. (2020). Neuronal and glial regulation of CNS angiogenesis and barriergenesis. Development.

[3] Nortley R., Attwell D. (2017). Control of brain energy supply by astrocytes. Curr. Opin. Neurobiol..

[4] Longden T.A., Hill-Eubanks D.C., Nelson M.T. (2016). Ion channel networks in the control of cerebral blood flow. J. Cereb. Blood Flow Metab..

[5] Mishra A. (2017). Binaural blood flow control by astrocytes: Listening to synapses and the vasculature. J. Physiol..

[6] Attwell D., Buchan A.M., Charpak S., Lauritzen M., Macvicar B.A., Newman E.A. (2010). Glial and neuronal control of brain blood flow. Nature.

[7] Huang S.Y., Sung C.S., Chen W.F., Chen C.H., Feng C.W., Yang S.N., Hung H.C., Chen N.F., Lin P.R., Chen S.C. (2015). Involvement of phosphatase and tensin homolog deleted from chromosome 10 in rodent model of neuropathic pain. J. Neuroinflamm..

[8] Sofroniew M.V., Vinters H.V. (2010). Astrocytes: Biology and pathology. Acta Neuropathol..

[9] Li T., Chen X., Zhang C., Zhang Y., Yao W. (2019). An update on reactive astrocytes in chronic pain. J. Neuroinflamm..

[10] Doolen S., Iannitti T., Donahue R.R., Shaw B.C., Grachen C.M., Taylor B.K. (2018). Fingolimod reduces neuropathic pain behaviors in a mouse model of multiple sclerosis by a sphingosine-1 phosphate receptor 1-dependent inhibition of central sensitization in the dorsal horn. Pain.

[11] Gruber-Schoffnegger D., Drdla-Schutting R., Honigsperger C., Wunderbaldinger G., Gassner M., Sandkuhler J. (2013). Induction of thermal hyperalgesia and synaptic long-term potentiation in the spinal cord lamina I by TNF-alpha and IL-1beta is mediated by glial cells. J. Neurosci..

[12] Watkins L.R., Maier S.F. (2003). Glia: A novel drug discovery target for clinical pain. Nat. Rev. Drug Discov..

[13] Ji R.R., Nackley A., Huh Y., Terrando N., Maixner W. (2018). Neuroinflammation and Central Sensitization in Chronic and Widespread Pain. Anesthesiology.

[14] Gantenbein A.R., Sandor P.S. (2006). Physiological parameters as biomarkers of migraine. Headache.

[15] Montoro C.I., Duschek S., de Guevara C.M., Reyes Del Paso G.A. (2016). Patterns of Cerebral Blood Flow Modulation During Painful Stimulation in Fibromyalgia: A Transcranial Doppler Sonography Study. Pain Med..

[16] Ingber D., Fujita T., Kishimoto S., Sudo K., Kanamaru T., Brem H., Folkman J. (1990). Synthetic analogues of fumagillin that inhibit angiogenesis and suppress tumour growth. Nature.

[17] Folkman J., Shing Y. (1992). Angiogenesis. J. Biol. Chem..

[18] Folkman J. (2002). Role of angiogenesis in tumor growth and metastasis. Semin. Oncol..

[19] Seabrook T.J., Littlewood-Evans A., Brinkmann V., Pollinger B., Schnell C., Hiestand P.C. (2010). Angiogenesis is present in experimental autoimmune encephalomyelitis and pro-angiogenic factors are increased in multiple sclerosis lesions. J. Neuroinflamm..

[20] Girolamo F., Coppola C., Ribatti D., Trojano M. (2014). Angiogenesis in multiple sclerosis and experimental autoimmune encephalomyelitis. Acta Neuropathol. Commun..

[21] Johnson E.A., Guignet M.A., Dao T.L., Hamilton T.A., Kan R.K. (2015). Interleukin-18 expression increases in response to neurovascular damage following soman-induced status epilepticus in rats. J. Inflamm..

[22] Kirk S.L., Karlik S.J. (2003). VEGF and vascular changes in chronic neuroinflammation. J. Autoimmun..

[23] Paulson P.E., Casey K.L., Morrow T.J. (2002). Long-term changes in behavior and regional cerebral blood flow associated with painful peripheral mononeuropathy in the rat. Pain.

[24] Paulson P.E., Morrow T.J., Casey K.L. (2000). Bilateral behavioral and regional cerebral blood flow changes during painful peripheral mononeuropathy in the rat. Pain.

[25] Hu X.M., Yang W., Du L.X., Cui W.Q., Mi W.L., Mao-Ying Q.L., Chu Y.X., Wang Y.Q. (2019). Vascular Endothelial Growth Factor A Signaling Promotes Spinal Central Sensitization and Pain-related Behaviors in Female Rats with Bone Cancer. Anesthesiology.

[26] Lin J., Li G., Den X., Xu C., Liu S., Gao Y., Liu H., Zhang J., Li X., Liang S. (2010). VEGF and its receptor-2 involved in neuropathic pain transmission mediated by P2X(2)(/)(3) receptor of primary sensory neurons. Brain Res. Bull..

[27] Guruceaga X., Perez-Cuesta U., Abad-Diaz de Cerio A., Gonzalez O., Alonso R.M., Hernando F.L., Ramirez-Garcia A., Rementeria A. (2019). Fumagillin, a Mycotoxin of Aspergillus fumigatus: Biosynthesis, Biological Activities, Detection, and Applications. Toxins.

[28] Zitvogel L., Apetoh L., Ghiringhelli F., Kroemer G. (2008). Immunological aspects of cancer chemotherapy. Nat. Rev. Immunol..

[29] Dasgupta B., Yi Y., Hegedus B., Weber J.D., Gutmann D.H. (2005). Cerebrospinal fluid proteomic analysis reveals dysregulation of methionine aminopeptidase-2 expression in human and mouse neurofibromatosis 1-associated glioma. Cancer Res..

[30] Ballabh P., Braun A., Nedergaard M. (2004). The blood-brain barrier: An overview: Structure, regulation, and clinical implications. Neurobiol. Dis..

[31] Lin Y.C., Huang S.Y., Jean Y.H., Chen W.F., Sung C.S., Kao E.S., Wang H.M., Chakraborty C., Duh C.Y., Wen Z.H. (2011). Intrathecal lemnalol, a natural marine compound obtained from Formosan soft coral, attenuates nociceptive responses and the activity of spinal glial cells in neuropathic rats. Behav. Pharmacol..

[32] Sung C.S., Cherng C.H., Wen Z.H., Chang W.K., Huang S.Y., Lin S.L., Chan K.H., Wong C.S. (2012). Minocycline and fluorocitrate suppress spinal nociceptive signaling in intrathecal IL-1beta-induced thermal hyperalgesic rats. Glia.

[33] Chen N.F., Chen W.F., Sung C.S., Lu C.H., Chen C.L., Hung H.C., Feng C.W., Chen C.H., Tsui K.H., Kuo H.M. (2016). Contributions of p38 and ERK to the antinociceptive effects of TGF-beta1 in chronic constriction injury-induced neuropathic rats. J. Headache Pain.

[34] Chaplan S.R., Bach F.W., Pogrel J.W., Chung J.M., Yaksh T.L. (1994). Quantitative assessment of tactile allodynia in the rat paw. J. Neurosci. Methods.

[35] Haroutiunian S., Kagan L., Yifrach-Damari I., Davidson E., Ratz Y., Hoffman A. (2014). Enhanced antinociceptive efficacy of epidural compared with i.v. methadone in a rat model of thermal nociception. Br. J. Anaesth..

[36] Allbutt H.N., Henderson J.M. (2007). Use of the narrow beam test in the rat, 6-hydroxydopamine model of Parkinson’s disease. J. Neurosci. Methods.

[37] Sung C.S., Wen Z.H., Feng C.W., Chen C.H., Huang S.Y., Chen N.F., Chen W.F., Wong C.S. (2017). Potentiation of spinal glutamatergic response in the neuron-glia interactions underlies the intrathecal IL-1beta-induced thermal hyperalgesia in rats. CNS Neurosci. Ther..

[38] Ramos K.M., Lewis M.T., Morgan K.N., Crysdale N.Y., Kroll J.L., Taylor F.R., Harrison J.A., Sloane E.M., Maier S.F., Watkins L.R. (2010). Spinal upregulation of glutamate transporter GLT-1 by ceftriaxone: Therapeutic efficacy in a range of experimental nervous system disorders. Neuroscience.

[39] Kim C.E., Kim Y.K., Chung G., Im H.J., Lee D.S., Kim J., Kim S.J. (2014). Identifying neuropathic pain using (18)F-FDG micro-PET: A multivariate pattern analysis. Neuroimage.

[40] Holland-Fischer P., Greisen J., Grofte T., Jensen T.S., Hansen P.O., Vilstrup H. (2009). Increased energy expenditure and glucose oxidation during acute nontraumatic skin pain in humans. Eur. J. Anaesthesiol..

[41] Watanabe K., Hirano S., Kojima K., Nagashima K., Mukai H., Sato T., Takemoto M., Matsumoto K., Iimori T., Isose S. (2018). Altered cerebral blood flow in the anterior cingulate cortex is associated with neuropathic pain. J. Neurol. Neurosurg. Psychiatry.

[42] Carmeliet P. (2005). Angiogenesis in life, disease and medicine. Nature.

[43] Randi A.M., Laffan M.A. (2017). Von Willebrand factor and angiogenesis: Basic and applied issues. J. Thromb. Haemost..

[44] Chaudhary P., Marracci G.H., Bourdette D.N. (2006). Lipoic acid inhibits expression of ICAM-1 and VCAM-1 by CNS endothelial cells and T cell migration into the spinal cord in experimental autoimmune encephalomyelitis. J. Neuroimmunol..

[45] Pendu R., Terraube V., Christophe O.D., Gahmberg C.G., de Groot P.G., Lenting P.J., Denis C.V. (2006). P-selectin glycoprotein ligand 1 and beta2-integrins cooperate in the adhesion of leukocytes to von Willebrand factor. Blood.

[46] Woodfin A., Voisin M.B., Nourshargh S. (2007). PECAM-1: A multi-functional molecule in inflammation and vascular biology. Arterioscler. Thromb. Vasc. Biol..

[47] Park S., Sorenson C.M., Sheibani N. (2015). PECAM-1 isoforms, eNOS and endoglin axis in regulation of angiogenesis. Clin. Sci..

[48] Widenfalk J., Lipson A., Jubran M., Hofstetter C., Ebendal T., Cao Y., Olson L. (2003). Vascular endothelial growth factor improves functional outcome and decreases secondary degeneration in experimental spinal cord contusion injury. Neuroscience.

[49] Walsh D.A., McWilliams D.F., Turley M.J., Dixon M.R., Franses R.E., Mapp P.I., Wilson D. (2010). Angiogenesis and nerve growth factor at the osteochondral junction in rheumatoid arthritis and osteoarthritis. Rheumatology.

[50] Ashraf S., Mapp P.I., Walsh D.A. (2011). Contributions of angiogenesis to inflammation, joint damage, and pain in a rat model of osteoarthritis. Arthritis Rheum..

[51] Nagai T., Sato M., Kobayashi M., Yokoyama M., Tani Y., Mochida J. (2014). Bevacizumab, an anti-vascular endothelial growth factor antibody, inhibits osteoarthritis. Arthritis Res. Ther..

[52] Mapp P.I., Walsh D.A. (2012). Mechanisms and targets of angiogenesis and nerve growth in osteoarthritis. Nat. Rev. Rheumatol..

[53] Hamilton J.L., Nagao M., Levine B.R., Chen D., Olsen B.R., Im H.J. (2016). Targeting VEGF and Its Receptors for the Treatment of Osteoarthritis and Associated Pain. J. Bone Miner. Res.

[54] Itoh Y., Toriumi H., Yamada S., Hoshino H., Suzuki N. (2011). Astrocytes and pericytes cooperatively maintain a capillary-like structure composed of endothelial cells on gel matrix. Brain Res..

[55] Zhang S., Kim B., Zhu X., Gui X., Wang Y., Lan Z., Prabhu P., Fond K., Wang A., Guo F. (2020). Glial type specific regulation of CNS angiogenesis by HIFalpha-activated different signaling pathways. Nat. Commun..

[56] Huang L., Nakamura Y., Lo E.H., Hayakawa K. (2019). Astrocyte Signaling in the Neurovascular Unit After Central Nervous System Injury. Int. J. Mol. Sci..

[57] Vaillancourt M., Chia P., Medzikovic L., Cao N., Ruffenach G., Younessi D., Umar S. (2019). Experimental Pulmonary Hypertension Is Associated With Neuroinflammation in the Spinal Cord. Front. Physiol..

[58] Zhong Y., Chen J., Chen J., Chen Y., Li L., Xie Y. (2019). Crosstalk between Cdk5/p35 and ERK1/2 signalling mediates spinal astrocyte activity via the PPARgamma pathway in a rat model of chronic constriction injury. J. Neurochem..

[59] Zanjani T.M., Sabetkasaei M., Karimian B., Labibi F., Farokhi B., Mossafa N. (2010). The attenuation of pain behaviour and serum interleukin-6 concentration by nimesulide in a rat model of neuropathic pain. Scand. J. Pain.

[60] Chen J.Y., Chu L.W., Cheng K.I., Hsieh S.L., Juan Y.S., Wu B.N. (2018). Valproate reduces neuroinflammation and neuronal death in a rat chronic constriction injury model. Sci. Rep..

[61] Amin B., Hajhashemi V., Hosseinzadeh H., Abnous K. (2012). Antinociceptive evaluation of ceftriaxone and minocycline alone and in combination in a neuropathic pain model in rat. Neuroscience.

[62] Jancalek R., Dubovy P., Svizenska I., Klusakova I. (2010). Bilateral changes of TNF-alpha and IL-10 protein in the lumbar and cervical dorsal root ganglia following a unilateral chronic constriction injury of the sciatic nerve. J. Neuroinflamm..

[63] Barzelay A., Ben-Shoshan J., Entin-Meer M., Maysel-Auslender S., Afek A., Barshack I., Keren G., George J. (2010). A potential role for islet-1 in post-natal angiogenesis and vasculogenesis. Thromb. Haemost..

[64] Sethi G., Sung B., Aggarwal B.B. (2008). TNF: A master switch for inflammation to cancer. Front. Biosci..

