# Supplementary material for: Fumagillin Attenuates Spinal Angiogenesis, Neuroinflammation, and Pain in Neuropathic Rats after Chronic Constriction Injury
Source: Biomedicines. 2021 Sep 10;9(9):1187. doi: 10.3390/biomedicines9091187 (PMC8470034; doi:10.3390/biomedicines9091187)
Supplement: Supplementary file 1 [file biomedicines-09-01187-s001.zip › biomedicines-1340959-Supplementary material.pdf]

Supplementary materials:

Fumagillin attenuates spinal angiogenesis, neuroinflammation, and pain in neuropathic rats after chronic constriction injury

Zhi-Hong Wen[1,2,†], Shi-Ying Huang[3,†], Hsiao-Mei Kuo[1,4,†], Chao-Ting Chen[1], Nan-Fu Chen[5,6], Wu-Fu Chen[1,7], Kuan-Hao Tsui[8,9], Hsin-Tzu Liu[10] and Chun-Sung Sung[11,12,*]

[1]Department of Marine Biotechnology and Resources, National Sun Yat-Sen University., Kaohsiung, Taiwan; wzh@mail.nsysu.edu.tw (Z.-H. W.); cct337@gmail.com (C.-T. C.)

[2]Institute of BioPharmaceutical Sciences, National Sun Yat-sen University, Taiwan

[3]College of Food and Biological Engineering, Jimei University; Xiamen city, Fujian Province, China; johnhuang@jmu.edu.cn and johnjohnkings@gmail.com (S.-Y. H.)

[4]Center for Neuroscience, National Sun Yat-sen University, Kaohsiung, Taiwan; hsiaomeikuo@gmail.com (H.-M. K.)

[5]Division of Neurosurgery, Department of Surgery, Kaohsiung Armed Forces General Hospital, Kaohsiung, Taiwan; k802chen@gmail.com (N.-F. C.)

[6]Institute of Medical Science and Technology, National Sun Yat-sen University, Kaohsiung, Taiwan

[7]Department of Neurosurgery, Kaohsiung Chang Gung Memorial Hospital and Chang Gung University College of Medicine, Kaohsiung, Taiwan; ma4949@adm.cgmh.org.tw (W.-F. C.)

[8]Department of Obstetrics and Gynecology, Kaohsiung Veterans General Hospital, Kaohsiung, Taiwan; khtsui60@gmail.com (K.-H. T.)

[9]Institute of Clinical Medicine, School of Medicine, National Yang Ming Chiao Tung University, Taipei, Taiwan

[10]Department of Medical Research, Hualien Tzu Chi Hospital, Buddhist Tzu Chi Medical Foundation, Hualien, Taiwan; hsintzu_liu@tzuchi.com.tw (H.-T. L.)

[11]Division of Pain Management, Department of Anesthesiology, Taipei Veterans General Hospital, Taipei, Taiwan; cssung@vghtpe.gov.tw and sung6119@gmail.com (C.-S. S.)

[12]School of Medicine, National Yang Ming Chiao Tung University, Taipei, Taiwan

*Correspondence: cssung@vghtpe.gov.tw and sung6119@gmail.com; Tel: 886-2-28757549

†These authors contributed equally to this work.

Figure legends

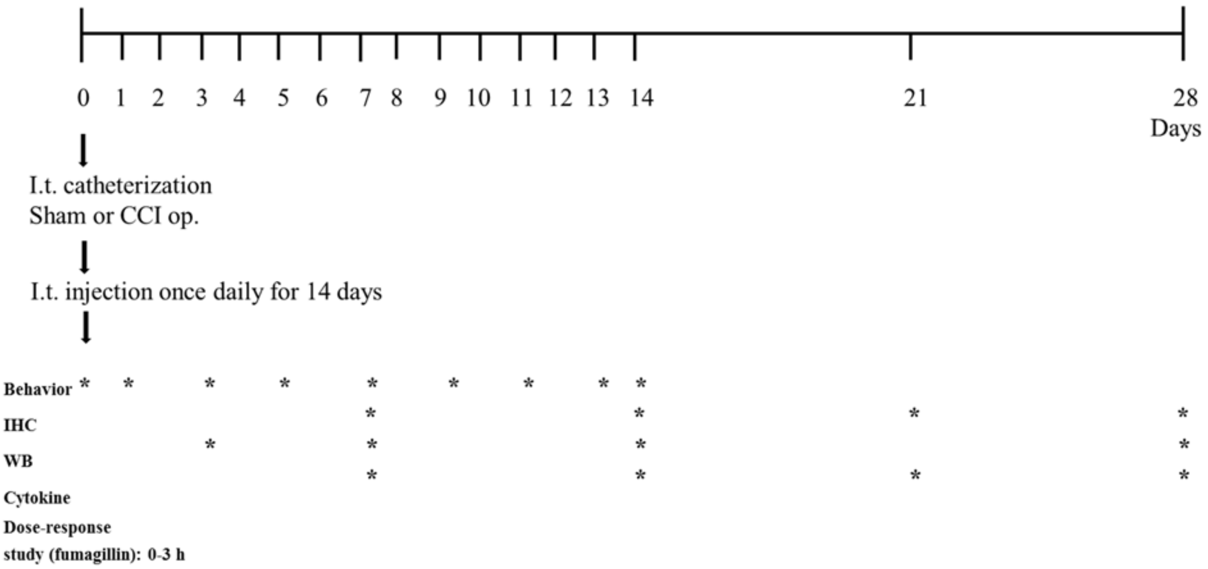

Figure S1: Experimental design. Schematic timeline of the experimental procedures. Abbreviations: CCI, chronic constriction injury; IHC, immunohistochemistry analysis; I.t., intrathecal; WB, Western blot analysis.

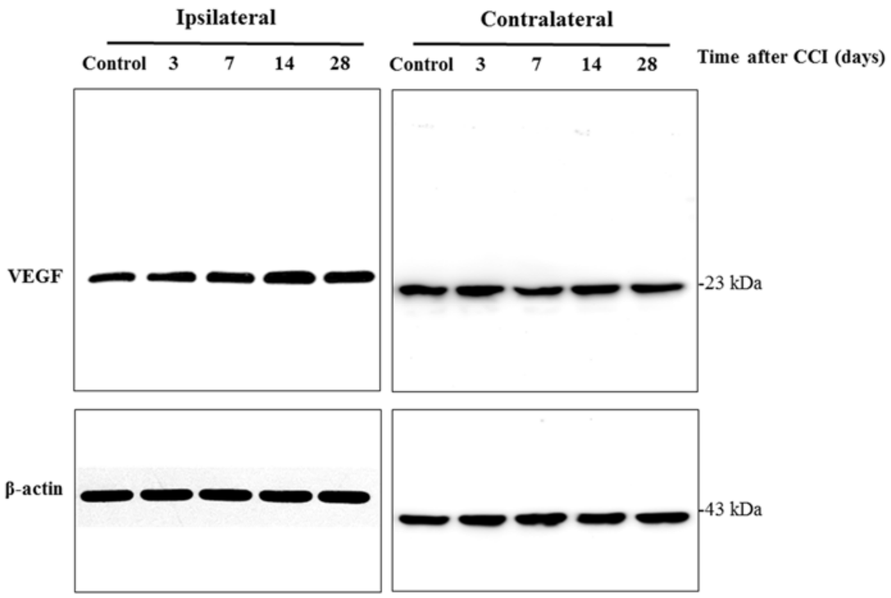

Figure S2: Representative uncropped images of Western Blots in Figure 1C: VEGF and β-actin.

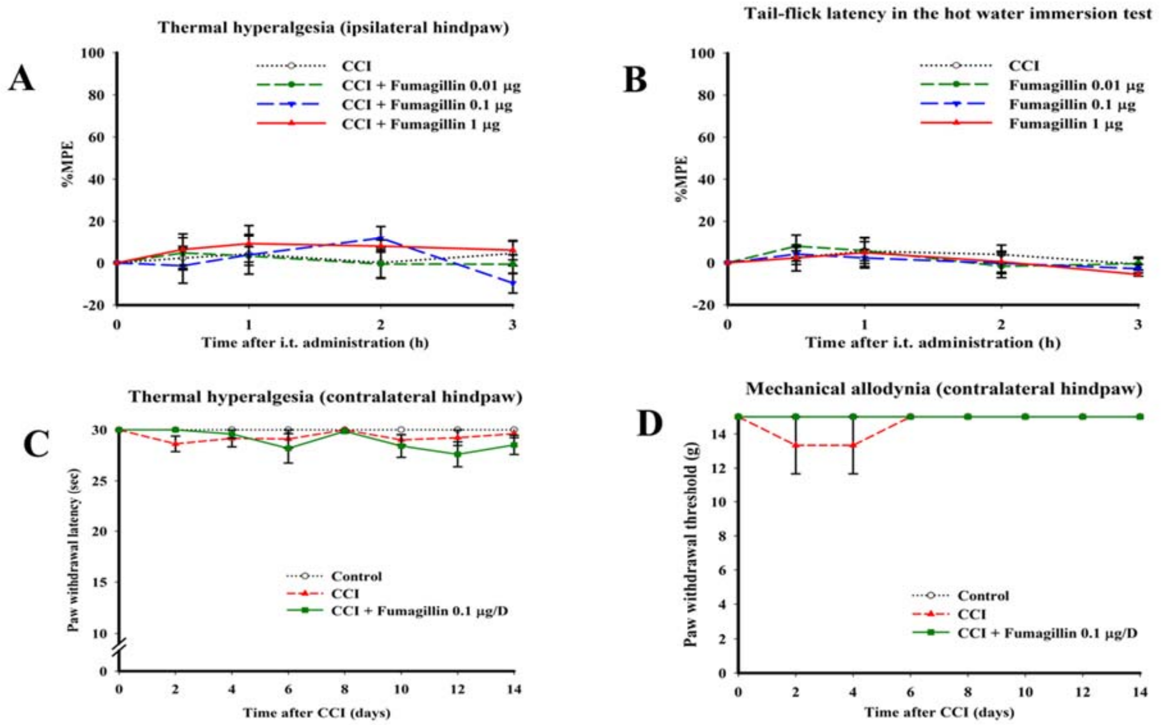

Figure S3: Effects of fumagillin intrathecal administration on the pain behaviors evoked by chronic constriction injury (CCI) in rats. Fumagillin *per se* had no analgesic effect in naïve and sham-operated rats for a 0.01–1 μg dose range. (A) Dose-response analysis of the effects of fumagillin single injection on CCI-induced thermal hyperalgesia to the radiant heat of the ipsilateral hind paw at postoperative day (POD) 14. (B) Dose-response analysis of the effects of fumagillin single injection on the tail-flick latency using the hot water immersion test in control and CCI rats at POD 14. The x-axis shows the time within 3 hours after drug injection, and the y-axis shows %MPE (maximal possible effect) calculated as a mean from three rats per dose. The area under the analgesic effect-time curve is the %MPE-time curve for intrathecal administration of 0.01, 0.1, and 1 μg of fumagillin. Intrathecal injection of fumagillin at a dose of 0.1 μg/day for 14 consecutive days does not affect the paw withdrawal latency in response to radiant heat (C) and the paw withdrawal threshold after stimulation with von Frey filaments (D) for the contralateral hind paw. All values were expressed as means ± standard error of means (SEMs) (n = 3 per group and each time point).

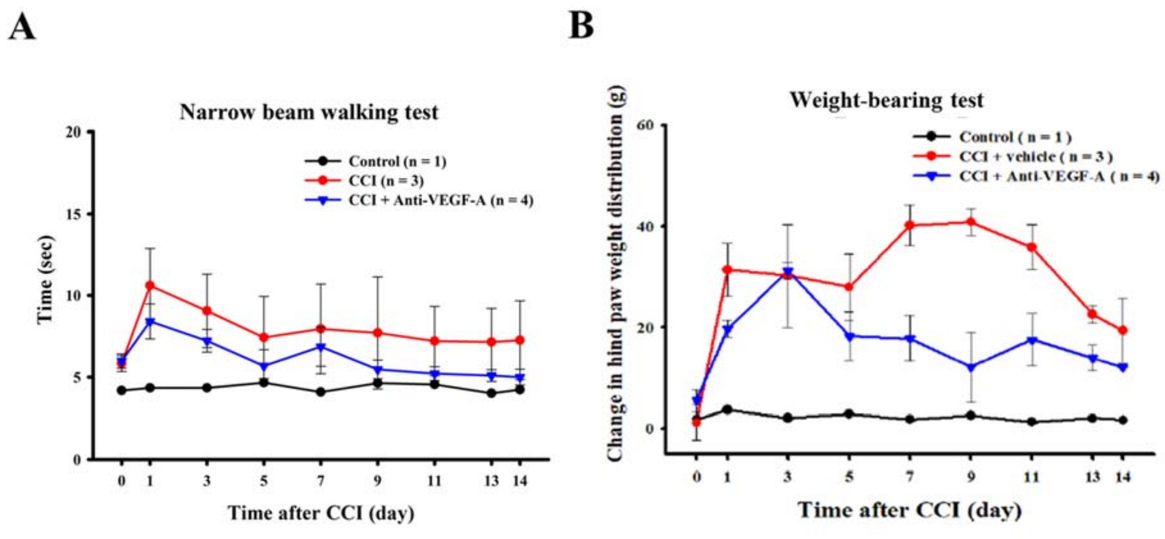

Figure S4: Effects of intrathecal administration of anti-vascular endothelial growth factor-A (anti-VEGF-A) monoclonal antibodies (0.3 μg/day) for 14 consecutive days on chronic constriction injury (CCI)-induced nociceptive behaviors. Anti-VEGF-A antibody has no neurotoxic effect on motor function, and all rats were free from motor disabilities with the i.t. administration of anti-VEGF-A antibody at a dose of 0.3 μg/day for 14 consecutive days. Narrow beam walking time and changes in hind paw weight distribution were measured in control rats intrathecally injected with artificial cerebrospinal fluid (aCSF) (control, n = 1), in CCI rats after intrathecal injection of aCSF (CCI or CCI + vehicle, n = 3), or anti-VEGF-A antibodies (CCI+ Anti-VEGF-A, n = 4). (A) The representative beam walking time of CCI + Anti-VEGF-A rats compares to CCI rats and control rat from POD 0–14. Anti-VEGF-A reduces the CCI-prolonged time to cross the beam. (B) The representative weight-bearing distribution of CCI + Anti-VEGF-A rats compares to CCI + Vehicle and control rats from POD 0–14. Anti-VEGF-A reduces the CCI-increased hind paw weight distribution. All values were expressed as means ± standard error of means (SEMs).
